# Application of CRISPR/Cas9-Based Reverse Genetics in *Leishmania braziliensis*: Conserved Roles for HSP100 and HSP23

**DOI:** 10.3390/genes11101159

**Published:** 2020-09-30

**Authors:** Vanessa Adaui, Constanze Kröber-Boncardo, Christine Brinker, Henner Zirpel, Julie Sellau, Jorge Arévalo, Jean-Claude Dujardin, Joachim Clos

**Affiliations:** 1 Bernhard Nocht Institute for Tropical Medicine, D-20359 Hamburg, Germany; vanessa.adaui@upc.edu.pe (V.A.); kroeber@bnitm.de (C.K.-B.); brinker@bnitm.de (C.B.); henner-444@gmx.net (H.Z.); sellau@bnitm.de (J.S.); 2 Instituto de Medicina Tropical Alexander von Humboldt, Universidad Peruana Cayetano Heredia, Lima 15102, Peru; jorge.arevalo@upch.pe; 3 Centre for Research and Innovation, Faculty of Health Sciences, Universidad Peruana de Ciencias Aplicadas, Lima 15067, Peru; JCDujardin@itg.be; 4 City of Hope National Medical Center, Duarte, CA 91010, USA; 5 Institute of Tropical Medicine, 2000 Antwerp, Belgium; 6 Department of Biomedical Sciences, University of Antwerp, 2000 Antwerp, Belgium

**Keywords:** *Leishmania braziliensis*, reverse genetics, CRISPR–Cas9, gene targeting, phenotyping, heat shock proteins

## Abstract

The protozoan parasite *Leishmania* (*Viannia*) *braziliensis (L. braziliensis*) is the main cause of human tegumentary leishmaniasis in the New World, a disease affecting the skin and/or mucosal tissues. Despite its importance, the study of the unique biology of *L. braziliensis* through reverse genetics analyses has so far lagged behind in comparison with Old World *Leishmania* spp. In this study, we successfully applied a cloning-free, PCR-based CRISPR–Cas9 technology in *L. braziliensis* that was previously developed for Old World *Leishmania major* and New World *L. mexicana* species. As proof of principle, we demonstrate the targeted replacement of a transgene (*eGFP*) and two *L. braziliensis* single-copy genes (*HSP23* and *HSP100*). We obtained homozygous Cas9-free *HSP23*- and *HSP100*-null mutants in *L. braziliensis* that matched the phenotypes reported previously for the respective *L. donovani* null mutants. The function of *HSP23* is indeed conserved throughout the Trypanosomatida as *L. major* *HSP23* null mutants could be complemented phenotypically with transgenes from a range of trypanosomatids. In summary, the feasibility of genetic manipulation of *L. braziliensis* by CRISPR–Cas9-mediated gene editing sets the stage for testing the role of specific genes in that parasite’s biology, including functional studies of virulence factors in relevant animal models to reveal novel therapeutic targets to combat American tegumentary leishmaniasis.

## 1. Introduction

The protozoan parasite *Leishmania* (*Viannia*) *braziliensis* (henceforth: *L. braziliensis)* is the main causative agent of human tegumentary leishmaniasis in Latin America. Infection with *L. braziliensis* generally causes cutaneous lesions, with possible, severe, metastatic mucosal involvement, and it is difficult to cure with the first-line pentavalent antimonial drugs [1,2,3,4]. In spite of its importance, the biology of *L. braziliensis* has not been analysed extensively, in part due to the limited set of genetic manipulation tools developed or adapted to this species. 

While Gene replacement using homologous recombination has proven a useful tool for testing gene function in Old World *Leishmania* spp. [5,6,7], yet—to our knowledge—no gene replacement analyses have been reported for *L. braziliensis*. However, a functional RNA interference (RNAi) machinery, predicted from the *L. braziliensis* genome sequence [8], was corroborated experimentally [9], allowing gene function analysis in this species [9,10]. The RNAi pathway and associated genes are absent in species of the *L*. (*Leishmania*) subgenus such as *L. major* and *L. donovani* [9]. However, RNAi-based gene knock-down is prone to off-target effects [11], which can confound phenotypic analyses.

Recently, the CRISPR (clustered regularly interspaced short palindromic repeats)–Cas9 (CRISPR-associated protein 9) technology is revolutionizing gene function studies in a wide range of organisms, due to its high efficiency, precision, relative simplicity, and versatility [12]. Using this tool, the Cas9 endonuclease can be directed to a specific genomic locus by a single guide RNA (sgRNA) to introduce a double-stranded break (DSB) in the target DNA [13]. DSBs compromise genomic integrity and are identified and repaired by the nuclear machinery by regulated and error-prone DNA repair pathways [14], and homologous donor DNA templates may be inserted introducing defined changes into the DNA near the DSB as part of the repair process [15]. 

CRISPR–Cas9-mediated gene targeting and gene editing (e.g., to generate point mutations, or add tags to endogenous genes) have been successfully developed and applied in kinetoplastids, including *Trypanosoma cruzi* [16], *T. brucei* [17,18], and several species of *Leishmania* [17,19,20,21,22,23,24], with the notable exception of New World *L*. (*Viannia*) species. This new technology has greatly improved the efficiency of gene targeting in *Leishmania* spp. over traditional homologous recombination-based gene replacement. 

First, CRISPR–Cas9 allows the rapid generation of gene deletion or gene disruption mutants in the promastigote stage (within 1–2 weeks depending on the species); thus minimising the occurrence of compensatory adaptations in the parasites [21,25]. This is particularly the case when a gene required for optimal *in vitro* survival and/or growth is targeted [26], since *Leishmania* have the remarkable ability to adapt to environmental changes by chromosome copy number variations [27,28]. Second, the generation of CRISPR-derived null mutants is facilitated by the use of donor DNA repair cassettes (containing antibiotic selection markers) flanked by short homology arms targeting the gene of interest (GOI), in a single transfection [17,29]. Third, both single and multigene families can be targeted with this system [16,30], and it even allows simultaneous editing of multiple loci [24,30], as well as the identification of essential genes [20,30,31]. CRISPR gene editing also allows for *in situ* addition of flanking loxP sites to a gene of interest and the subsequent rapamycin-inducible gene deletion by dimerisable Cre (DiCre) recombinase [32,33]. This facilitates deletion of essential genes and observation of the cell biological and morphological effects on living cells in a time-dependent manner.

In the absence of a donor DNA repair template, *Leishmania* use microhomology-mediated end-joining (MMEJ) or single-strand annealing (SSA) to repair DSBs, both of which lead to deletions of various sizes that disrupt the targeted gene [20,30,31]. These DSB repair pathways (MMEJ and SSA) have a generally low efficiency in *Leishmania*, and SSA may result in unwanted deletions of adjacent genes [31]. Transfections of a donor DNA template to facilitate homology-directed repair significantly improves CRISPR–Cas9 gene targeting efficiency and specificity, and eases the identification of CRISPR-edited mutants in *Leishmania* [17,19,20,30,31]. 

In this study, we establish the CRISPR–Cas9 technology as an experimental tool for reverse genetics in *L. braziliensis* facilitating the generation of null mutants and the analysis of gene function in this important human pathogen. We applied a cloning-free, PCR-based CRISPR–Cas9 method that was used successfully in *Leishmania mexicana*, *L. major*, *L. donovani*, and *Trypanosoma brucei* for rapid and precise gene editing [17,21]. As a proof of principle, we first targeted an integrated transgene coding for enhanced green fluorescent protein (eGFP) and then replaced two single-copy genes of *L. braziliensis* encoding heat shock proteins HSP23 and HSP100. In addition, we show that functions of these genes are conserved in the *Viannia* subgenus of *Leishmania.*

## 2. Materials and Methods

### 2.1. Leishmania Strains and Culture 

Promastigotes of the Peruvian *L. braziliensis* strain PER005 (MHOM/PE/01/LH2182(PER005)) [34] clone 2 (clone originally derived from a clinical isolate), *L. donovani* 1S (MHOM/SD/62/1S) [35], *L. major* 5-ASKH (MHOM/SU/73/5-ASKH) [36], and their genetically modified derived lines reported in this study were routinely grown at 25 °C in monophasic M199 medium (Sigma-Aldrich, München, Germany) supplemented with 20% heat-inactivated fetal calf serum (Sigma-Aldrich), 10 mg/L hemin, 100 μM adenine, 5 μM 6-biopterin, 40 mM HEPES (pH 7.4), 2 mM L-glutamine, 100 units/ml penicillin and 100 μg/mL streptomycin (hereafter referred as complete M199 medium) [37,38]. Cultures were subcultured to fresh medium every 3–4 days. Appropriate selection drugs were added to the medium when necessary as indicated below. The isolation and use of ex vivo macrophage progenitor cells from mice was duly registered with the Animal Protection Authority of the State of Hamburg and in accordance with the German Animal Protection Law.

### 2.2. Promastigote Cultivation

Promastigotes were grown in complete M199 medium in 25 cm^2^ cell culture flasks. Cell density was monitored using a CASY^®^ Cell Counter and Analyzer (Roche, Mannheim, Germany).

### 2.3. Transfections, Selection, and Cell Cloning

Electrotransfection of circular DNA was performed using a Bio-Rad Gene Pulser apparatus and electroporation conditions as described [39]. Briefly, promastigotes grown to mid-log phase were harvested by centrifugation (1251 *g*, 10 min, 4 °C), washed twice with ice-cold phosphate-buffered saline (PBS), once in pre-chilled electroporation buffer, and suspended in electroporation buffer at a density of 1 × 10^8^ parasites/mL. 

For the generation of double allele replacements and for the integration of linearised DNA constructs, cells were transfected following the Amaxa protocol as described previously [17,40]. Briefly, 1 × 10^7^ promastigotes grown to mid- to late-log phase were harvested by centrifugation at 1251 *g* for 10 min (at RT), washed once with 1 × Tb-BSF electroporation buffer (90 mM NaHPO_3_, 5 mM KCl, 0.15 mM CaCl_2_, 50 mM HEPES, pH 7.3) [41] at RT, and suspended in 150 μL electroporation buffer per transfection. For gene editing, the cell suspension was mixed with the pooled unpurified PCR amplicons for the two single-guide RNA (sgRNA) templates and two donor DNAs (combined volume approximately 100 μL, heat-sterilised at 94 °C for 5 min before transfection) in a total volume of 250 μL. For integration of transgenes into the 18S SSU rRNA locus, cells were mixed with 2 µg of the *Swa*I-linearised DNA construct. Electroporation was performed in a 0.2 cm gap Gene Pulser electroporation cuvette (Bio-Rad, München, Germany) using one pulse with program X-001 in the Amaxa Nucleofector IIb device (Lonza, Basel, Switzerland). A mock transfection control without DNA was included to check the real transfection efficiency.

Following electroporation, cells were immediately transferred into 5 mL drug-free pre-warmed complete M199 medium in 25 cm^2^ cell culture flasks. After parasite recovery at 25 °C for 16–20 h, the selection antibiotics were added at the indicated strain-specific concentrations. Nourseothricine (ClonNat, at 150 μg/mL for all parasite species; Werner BioAgents, Jena, Germany), hygromycin B (at 50 μg/mL for all parasite species; Roth, Karlsruhe, Germany), bleocin (at 5 μg/mL; Calbiochem, San Diego, CA, USA). Additionally, blasticidin (at 10 μg/mL for *L. donovani* and *L. major*; at 2.5 μg/mL for *L. braziliensis*; Roth, Karlsruhe, Germany) and puromycin (at 25 μg/mL for *L. donovani* and *L. major*; at 10 μg/mL for *L. braziliensis*; Sigma-Aldrich, München, Germany) were used to select for integration of the donor gene fragments. For *L. braziliensis*, double drug-resistant cell populations with the intended gene replacements were first selected at a lower selection pressure as indicated until they emerged in culture (about 2–3 weeks), followed by an increase in the selection pressure (at ~IC_99.7_: 5 μg/mL blasticidin; ~IC_96_: 20 μg/mL puromycin) to allow discrimination with the mock-transfected control cultures. 

For cloning by limiting dilution, exponential log-phase cultures of the candidate *L. braziliensis HSP23-* and *HSP100*-null mutants were seeded in complete M199 medium at 0.5 cells per well in two 96-well microtitre plates, as described previously [39]. After 14 days, monitoring of wells for promastigote growth by light microscopy was started and continued until growth-positive wells were observed. The contents of positive wells were seeded into 2 ml complete M199 medium maintaining the drug pressure (blasticidin and puromycin at ~IC_96_–IC_99.7_) in 25 cm^2^ cell culture flasks to expand the culture. Each population that emerged from an individual well was considered an individual clone.

### 2.4. Construction and Preparation of Recombinant DNA

HSP23-encoding genes of different kinetoplastid species including *L. donovani* (LdBPK_340230), *L. major* (LmjF.34.0210), *L. infantum* (LinJ.34.0230), *L. braziliensis* (LbrM.20.0220), *Trypanosoma brucei* (Tb927.10.2620), were amplified from species-specific genomic DNA using primer pairs that introduce a *Kpn*I and a *Bcl*I or *BamH*I (for *L. infantum* only) restriction sites (Table S1). Fragments were subsequently ligated into the *Leishmania* expression plasmid pCL1S [42] previously digested with *Kpn*I and *Bgl*II.

### 2.5. PCR-Amplification of Targeting Constructs

For gene disruption in *L. braziliensis*, PCR amplification of sgRNA templates (using a common sgRNA scaffold primer) and of donor DNAs, the latter from pTBlast and pTPuro plasmids [17], was done using the Expand^TM^ High Fidelity PCR System (Roche, Mannheim, Germany) and PCR conditions as described [40].

For gene disruption in *L. major*, sgRNA templates were amplified in a total volume of 20 µL using 1 × iProof high-fidelity PCR master mix (Bio-Rad, München, Germany), 2 µM G00 primer (sgRNA scaffold) and 2 µM LmHSP23-specific 3’sgRNA or 5’sgRNA primer (Table S1). Cycling conditions were 30 s at 98 °C followed by 35 cycles of 10 s at 98 °C, 30 s at 55 °C, 15 s at 72 °C, and a final elongation step of 10 min at 72 °C. The targeting fragments were amplified from 10 ng pTPuro or pTBlast plasmid in 1 × iProof mix (Bio-Rad) using 2 µM forward and reverse primers, 3% DMSO in a total volume of 25 µL. PCR steps were 3 min at 98 °C followed by 35 cycles of 30 s at 98 °C, 30 s at 65 °C, 30 s at 72 °C, and a final elongation step of 5 min at 72 °C.

### 2.6. Analytical PCR 

To screen for target-gene disruption in drug-resistant transfectant cell lines, genomic DNA was isolated from non-clonal populations of *eGFP-*deletion mutants and analysed by PCR. Genomic DNA was isolated using ISOLATE II Genomic DNA Kit (Bioline, Luckenwalde, Germany). 

To test for the presence of the *eGFP* ORF and integration of the drug-resistance genes (*BSD*, blasticidin-S deaminase; and *PAC*, puromycin N-acetyltransferase) in the *eGFP* mutants, 1 μL of isolated DNA was mixed with 1 × iProof high-fidelity PCR master mix (Bio-Rad), 0.4 μM each forward and reverse primers, and 12% DMSO in a 25.5 µL total volume. In parallel, a technical control PCR (to demonstrate the presence of DNA in the analysed samples) was performed by amplifying a fragment from the *L. donovani HSP23* or *L. braziliensis actin* ORFs. PCR steps were 3 min at 98 °C followed by 30 cycles of 30 s at 98 °C, 30 s at 60 °C, 30 s at 72 °C followed by a final elongation step for 5 min at 72 °C.

The *Leishmania* wild-type and parental cell lines were included as controls. 10 µL of each PCR reaction was run on a 1% agarose gel to check for the presence of the expected product. The list of primer pairs used is given in Table S1. 

### 2.7. RNA Extraction, cDNA Synthesis, and Quantitative Real-Time PCR (qRT-PCR)

qRT-PCR was performed essentially as described [43]. Total RNA was isolated from 5 × 10^7^ parasites using the InviTrap spin cell RNA mini kit (STRATEC Molecular GmbH, Berlin, Germany) according to manufacturer’s instructions. First strand cDNA synthesis was performed using a mix of oligo-dT and random primers (QuantiTect Reverse Transcription kit, Qiagen, Hilden, Germany) following the manufacturer’s protocol. Real-time qPCR reactions were performed in a 20 μL-reaction mixture consisting of 1 μL of cDNA sample, 0.5 μM each gene-specific forward and reverse primers, and 1 × DyNAmo Color Flash SYBR Green Master Mix (Thermo Fisher Scientific, Waltham, MA, USA). The primers used for amplification of the target and reference genes are listed in Table S1. Reactions were run on a Rotor-Gene^TM^ RG 3000 Instrument (Corbett, Sydney, Australia) using the following thermal cycling conditions: an initial denaturation step at 95 °C for 7 min, followed by 35 cycles at 95 °C for 15 s, 69 °C for 20 s, and 71 °C for 30 s. After PCR amplification, a step at 95 °C for 1 min was included, followed by a melting curve analysis (67–95 °C, hold 60 s on the first step, hold 8 s on next steps). Data collection and analysis were performed with the Rotor-Gene real-time analysis software 6.1.81 (Corbett, Sydney, Australia). The normalised expression ratio was calculated using the 2^–ΔΔ^^Cq^ method [44].

### 2.8. Next Generation Sequencing

DNA library construction, next generation sequencing and data analyses were performed as described [45]. Paired sequence data were aligned against a novel long-read assembly of the *L. braziliensis* M2904 reference genome [46].

### 2.9. Western Blotting

Western blots were performed following established protocols [38,47].

### 2.10. Immunofluorescence Assays 

Indirect immunofluorescence microscopy was performed as described [48]. 

### 2.11. Flow Cytometry Cell Analysis

For GFP quantification, 2 × 10^6^ parasites were harvested (1251 *g*, 10 min, 4 °C), washed once in PBS, fixed in 4% paraformaldehyde in PBS for 20 min at RT, washed twice in PBS, resuspended in 150 μL PBS, and immediately analysed by flow cytometry. The Cas9–GFP-expressing parental cell lines served as positive controls. The Cas9-expressing lines, which were negative for GFP, were included as negative controls to assess background fluorescence. Flow cytometric measurements were performed with the Accuri^TM^ C6 flow cytometer (BD Biosciences, Heidelberg, Germany). A total of 30,000 events were recorded and analysed with FlowJo^TM^ software V 10 (Becton, Dickinson and Company, Ashland, OR, USA).

### 2.12. In Vitro Infection of Murine Bone Marrow-Derived Macrophages

In vitro infections and parasite load quantification were performed as described [49,50,51].

### 2.13. In Silico Procedures

In silico cloning, DNA and protein sequence analysis were performed using the MacVector software version 17.x (Mac Vector, Cambridge, United Kingdom). Post-acquisition processing of images was performed using the ImageJ Fiji Software (Version 2.0.0, https://fiji.sc). Composite figures for publication were prepared using the Intaglio software (Purgatory Design, Durango, CO, USA). Numerical data and statistical differences were analysed using Prism (version 8, GraphPad Software, San Diego, CA, USA). Statistical comparisons between groups in the promastigote growth experiments were conducted using one-way analysis of variance (ANOVA)/Kruskal–Wallis test with Dunn’s post test. For comparison of intracellular parasite survival within macrophages, a ratio-paired, one-sided Student’s *t*-test was applied to offset the variability between primary cell populations. Differences were considered significant at *p* < 0.05.

In silico design of primers to generate sgRNA templates and donor DNA was performed essentially as described [40]. Guide RNA sequences were designed using the Eukaryotic Pathogen CRISPR gRNA Design Tool (EuPaGDT, available at http://grna.ctegd.uga.edu) [52], using the default parameters (SpCas9: 20 nt gRNA length; PAM: NGG on 3’ end; off-target PAM: NAG, NGA). In addition, two guide RNA sequences targeting *eGFP* (*eGFP*-52-5’sgRNA and *eGFP*-553-5’sgRNA) were retrieved from the Addgene repository (deposited as gRNA1 and gRNA2 by Guigo, Johnson; available at https://www.addgene.org/search/all/) as they had been experimentally validated for use in CRISPR experiments. Target-specific sgRNA primers were then designed manually and contained the T7 promoter (for T7 RNA polymerase-driven in vivo transcription of the sgRNA), the 20 nt sgRNA target sequence, and a sequence complementary to the sgRNA scaffold [17].

To generate gene replacement mutants, target-specific sgRNA primers were produced at http://www.leishgedit.net [17] (for whole GOI disruption) or designed manually (for partial GOI disruption). Donor DNA primer sequences contained target-specific 30 nt homology flanks corresponding to sequences immediately adjacent to the sgRNA target sequence for DSB-mediated repair by homologous recombination and recognition sequences for the pT template plasmids and were generated at http://www.leishgedit.net (for whole GOI disruption) or designed manually (for partial GOI disruption).

Since the sgRNA and donor DNA sequences identified using the EuPaGDT and LeishGEdit online tools used the *L. braziliensis* reference genomes (M2904 and M2903) available in TriTrypDB (https://tritrypdb.org/tritrypdb/), we verified the specificity of each sgRNA and homology flanks (donor DNA) by alignment against the *L. braziliensis* PER005cl2 genome [46] (focussing on chromosomes 20 and 29 which harbour the genes of interest) using the MacVector™ software ( Mac Vector, Cambridge, United Kingdom). 

Oligonucleotides were ordered from Sigma-Aldrich (München, Germany). See Table S1 for a list of all primers. 

## 3. Results

### 3.1. Optimisation and Validation of the CRISPR-Cas9 System in L. braziliensis

**Figure 1:**
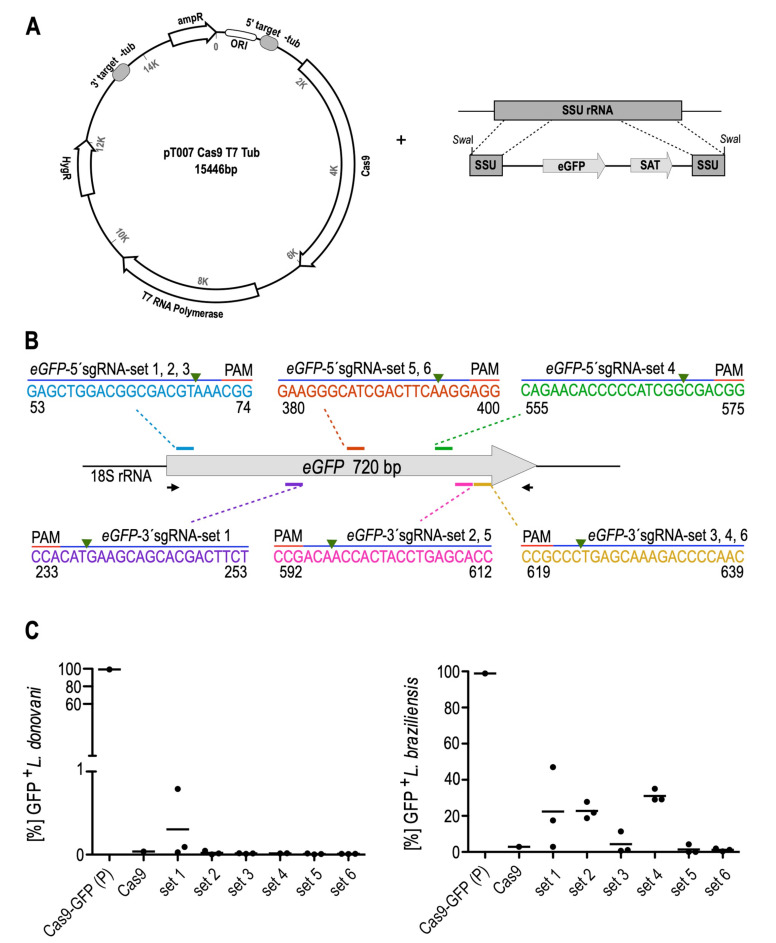
CRISPR–Cas9-mediated disruption of *eGFP* gene as proof-of-principle test in *L. braziliensis*. (**A**) Generation of Cas9–eGFP-expressing parasites. *Left panel*: plasmid pTB007 [17] bearing *hSpCas9* and *T7 RNAP* transgenes was transfected as circular episome into *L. braziliensis* PER005cl2 wild-type parasites. Transfectants were selected under Hygromycin B pressure. *Right panel*: schematic depiction of the double cross-over homologous recombination strategy to integrate the linearised pIR–*eGFP* construct into the SSU rRNA locus of *L. braziliensis* Cas9-expressing parasites. Regions shown are the SSU rRNA sequences on either ends resulting from *Swa*I restriction digest, the *eGFP* ORF, and the nourseothricine resistance gene ORF (*SAT*, encoding streptothricin-acetyltransferase). (**B**) Schematic representation of the *eGFP* locus and locations of the six 20-nt guide RNA sequences used for gene disruption; the guide sequence pairs with the DNA target (blue bar), directly upstream of a requisite 5’-NGG-3’ adjacent motif (PAM). The green arrowhead indicates the predicted Cas9 cleavage sites. Only the coding strand is shown. Binding sites of primers used for genotyping of genetically engineered parasites are denoted by arrows. The PCR fragment size depended on the pair of single guide RNAs (sgRNAs) tested. Sets of sgRNAs tested: set 1 = *eGFP*-52-5’sgRNA and *eGFP*-253-3’sgRNA; set 2 = *eGFP*-52-5’sgRNA and *eGFP*-612-3’sgRNA; set 3 = *eGFP*-52-5’sgRNA and *eGFP*-639-3’sgRNA; set 4 = *eGFP*-553-5’sgRNA and *eGFP*-639-3’sgRNA; set 5 = *eGFP*-378-5’sgRNA and *eGFP*-612-3’sgRNA; set 6 = *eGFP*-378-5’sgRNA and *eGFP*-639-3’sgRNA. (**C**) Flow cytometry analysis of eGFP–Cas9-expressing parasites before and after transfection of *eGFP*-targeting sgRNAs. Efficiency of *eGFP* disruption using 6 different sets of sgRNAs in *L. donovani* (left panel) and *L. braziliensis* (right panel) as quantified by GFP expression. Each set of two sgRNAs was co-transfected with two donor DNAs; transfections were done in triplicate. Sets of sgRNAs tested (labelled as set 1 to 6 in the graphs) consisted of pairs as described in Figure 1B. P, parental cell line Cas9/T7/eGFP. The gating scheme, a representative histogram, and all FACS plots showing the percentage of GFP-positive cells are shown in Supplemental Figures S2 and S3.

To test the feasibility and efficiency of sgRNA-guided, Cas9-mediated gene editing in *L. braziliensis*, we first targeted an integrated transgene coding for green fluorescent protein (eGFP). To this end, we generated a stable cell line of *L. braziliensis* expressing Cas9 and T7 RNAP from an episome (pTB007). The eGFP coding sequence was fused into the pIR-mcs3+ plasmid [53], and the linearised plasmid was transfected into *L. braziliensis*, leading to integration into the small subunit rRNA (18S) coding sequence (Figure 1A). 

We confirmed the expression of Cas9 protein by Western blot analysis (Figure S1A) and the detection of *T7RNAP* mRNA by qRT-PCR (Figure S1B). To better assess the efficiency of CRISPR–Cas9-mediated gene editing in *L. braziliensis*, we included Old World *L. donovani* strain 1S for comparative purposes, since the latter has long been used as a model for homologous recombination and genetic complementation in our laboratory. 

The *L. braziliensis* and *L. donovani* parental cell lines (Cas9/T7/GFP) were co-transfected with a pair of *eGFP*-targeted sgRNAs and corresponding donor DNA cassettes (i.e., homologous repair templates) to facilitate homology-directed repair [54,55]. Six different sets of dual sgRNAs and donor DNAs (Figure 1B; Table S1) were tested in triplicate. Transfectants were subjected to blasticidin and puromycin drug selection. At this point, drug selection (hygromycin B) for maintenance of the pTB007 episome encoding Cas9 and T7 RNAP and nourseothricine selection for the integrated pIR-mcs-*eGFP* were stopped.

In *L. donovani* 1S, the antibiotic selection pressure with the drug-selectable markers was kept constant throughout the selection period (10 μg/mL blasticidin, 25 μg/mL puromycin), following the optimised conditions established previously for this parasite strain in our group (data not shown). Survival of *L. donovani* double drug-resistant transfectants became apparent 6–10 days after transfection. Transfectants with *eGFP*-targeted sgRNAs set 5 and set 6 were the first to emerge in culture (6 and 9 days after transfection, respectively). Candidate *eGFP* replacement populations were passaged at least twice before analysing the gene disruption outcome by flow cytometry. Each of the 6 pairs of sgRNAs resulted in highly efficient reduction of GFP expression (Figure 1C, left panel; Figure S2). PCR analysis of genomic DNA with primers amplifying the entire *eGFP* ORF showed no detectable band corresponding to the *eGFP* transgene in all selected *L. donovani* lines, but bands of higher size appeared, indicating the integration of the donor repair cassettes (Figure S4B, left panel), as expected (Figure S4A). This was verified with *BSD* and *PAC* gene-specific primers (Figure S4B, left panel) and confirmed the high efficiency of CRISPR–Cas9-mediated *eGFP* disruption in *L. donovani*. 

In *L. braziliensis* PER005cl2 we first established the suitable concentrations of antibiotic selection through titration curves for 7 days (Figure S5). On this basis we decided to subject the parasites at first to the lowest concentrations of antibiotics that had a growth inhibitory effect, i.e., blasticidin at 2.5 μg/mL (~IC_85_) and puromycin at 10 μg/mL (~IC_65_). The first *L. braziliensis* drug-resistant transfectants to emerge in culture, as in *L. donovani*, were those transfected with *eGFP*-targeted sgRNAs set 5 (12–14 days after transfection) and set 6 (14 days after transfection). Transfectants with the other *eGFP* sgRNA sets (1, 2, 3 and 4) emerged 18–22 days after transfection. Candidate *eGFP* replacement populations were passaged at least twice and then analysed by flow cytometry as non-clonal populations. By flow cytometric analysis, sgRNAs sets 5 and 6 were the most efficient to abrogate the eGFP expression (0.02–4.30% GFP-positive cells), whereas sgRNA set 3 was slightly less efficient (0.69–11.4% GFP-positive cells). The sgRNAs sets 1, 2 and 4 were the least efficient (2.91–47.00% GFP-positive cells) (Figure 1C, right panel; Figure S3). Genomic DNAs from these parasite populations were examined by PCR confirming a complete loss of the *eGFP* transgene only in three selected *L. braziliensis* lines (*eGFP*-null mutants 5.1, 5.3 and 6.3) (not shown), which were transfected with the most potent sgRNAs, sets 5 and 6. For the other selected *L. braziliensis* lines, a band corresponding to the unmodified *eGFP* gene was still detected with varying intensities (Figure S4, right panel, for *eGFP* mutants 3.1, 3.2, and 3.3). PCR analysis with *eGFP* gene-specific primers also showed bands of higher size indicating the integration of the donor repair cassettes in the *L. braziliensis eGFP* mutants (Figure S4B, right panel), as expected (Figure S4A). While the blasticidin replacement cassette was confirmed to be integrated in all *L. braziliensis* selected lines by PCR analysis with *BSD*-specific primers (Figure S4B, right panel), the puromycin replacement cassette was detected in twelve out of 18 selected *L. braziliensis* lines, as assessed using *PAC*-specific primers (Figure S4B, right panel). This outcome reflected the moderate antibiotic selective pressure used to generate the *L. braziliensis eGFP* mutants.

At day 35 after transfection of the *L. braziliensis* Cas9/T7/eGFP parental cell line, inspection of the two *L. braziliensis* mock-transfected controls showed minimal growth. To impose a more stringent dual antibiotic selection, the mock cultures and selected *eGFP* mutants were passaged in complete M199 medium with blasticidin at 5 μg/mL (~IC_99.7_) and puromycin at 20 μg/mL (~IC_96_). The mock-transfected cultures succumbed to the antibiotic pressure within 4 days, while the *eGFP* mutant populations proliferated. This double antibiotic selection regimen was used in all subsequent experiments. 

### 3.2. CRISPR–Cas9-Mediated Disruption of Endogenous HSP23 and HSP100 Genes in L. braziliensis

Next, we tested the applicability of the PCR-based CRISPR–Cas9 method on two endogenous, single-copy genes of *L. braziliensis* encoding the heat shock proteins HSP23 and HSP100. Both genes were successfully replaced in Old World *Leishmania* spp, using homologous recombination, giving rise to conditional phenotypes [47,56,57]. Previous work in *L. donovani* showed that *HSP23* null mutants are sensitive to temperature and chemical stresses. In *L. major* and *L. donovani*, ∆*clpB* (*HSP100*) null mutants showed loss of virulence in vitro and in vivo. We sought to replicate those findings in *L. braziliensis* to assess the practical application of CRISPR–Cas9-mediated genetic manipulation in this parasite species. First, we tested the fitness of *L. braziliensis* (Cas9/T7) cells by *in vitro* growth analysis (Figure S1C) and found slightly increased proliferation compared with wild type cells, thus excluding overt, detrimental effects of Cas9 expression.

For disruption of each targeted GOI, the *L. braziliensis* Cas9/T7 parental cell line was transfected in parallel with four different sets of sgRNAs and donor DNAs (see Table S1 for nucleotide sequences). Double drug-resistant cell populations for both targeted genes emerged in culture at day 18 post transfection, and were then subjected to a higher drug selection pressure, as established for *eGFP* deletion. 

#### 3.2.1. *LbrHSP23* Gene Replacement

**Figure 2:**
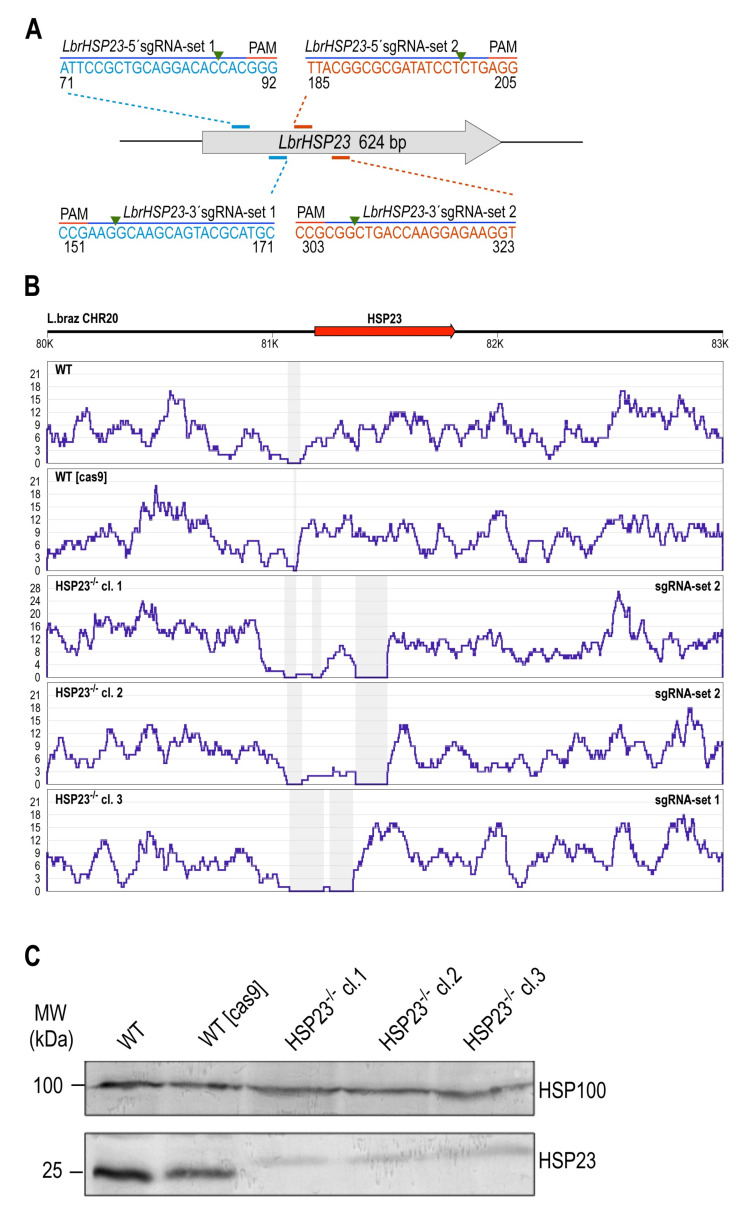
CRISPR–Cas9-mediated disruption of the endogenous *HSP23* gene in *L. braziliensis*. (**A**) Schematic representation of the *LbrHSP23* locus depicting the locations of 20-nt guide sequences that worked efficiently to disrupt the *LbrHSP23* ORF. Two sets of sgRNAs were tested (set 1 and set 2): set 1 = *LbrHSP23*-70-5’sgRNA and *LbrHSP23*-171-3’sgRNA; set 2 = *LbrHSP23*-183-5’sgRNA and *LbrHSP23*-323-3’sgRNA. Both pairs are designed to disrupt the conserved functional alpha-crystallin domain of HSP23 (amino acid positions 6–104). The guide sequence pairs with the DNA target (blue bar) directly upstream of a requisite 5′–NGG–3′ adjacent motif (PAM). The green arrowhead indicates the predicted Cas9 cleavage sites. Only the coding strand sequence is shown. (**B**) NGS analysis of the *HSP23* locus after CRISPR–Cas9-mediated gene replacement. Genomic DNA of *L. braziliensis* PER005cl2 wild-type parasites (WT), the parental cell line WT [Cas9] and *HSP23*^–/–^ mutant clones was isolated and subjected to NGS analysis. Resulting NGS reads were aligned to the *HSP23* gene locus (LbrM.20.0220) in the *L. braziliensis* M2904 reference genome using the Bowtie 2 algorithm. The read coverages (Y-axis) for the gene locus are shown in blue. The arrow represents the position and direction of the coding sequence. The X-axis numbering refers to the nucleotide position (bp) on chromosome 20. Grey-shaded areas denote lack of aligned reads. (**C**) Verification of *HSP23* gene replacement by Western blot analysis. 1 × 10^7^ cells of WT, WT [Cas9], and of 3 *HSP23*^–/–^ clones were lysed and the cell lysates were analysed by SDS-PAGE and Western blot using anti-HSP23 (1/500, lower panel). Anti-HSP100 (1/1000, upper panel) was used as loading control. MW = Molecular weight in kilodalton.

Three pairs of sgRNAs targeted different sites within the *LbrHSP23* ORF (Figure 2A), while a fourth pair of sgRNAs was designed to create DSBs upstream and downstream of the GOI coding region for whole-gene deletion (not shown).

Putative *HSP23-*null mutants were obtained with sgRNAs sets 1 and 2 (Figure 2A), both of which disrupted the alpha-crystallin domain of HSP23, a conserved signature feature of the small heat shock protein family [58]. Transfection with sgRNAs set 3, which targeted the C terminal part of LbrHSP23, did not generate viable cells after double selection. No *LbrHSP23* whole-gene deletion mutants could be obtained with sgRNAs set 4, either. Later analysis revealed a one-base pair mismatch between primer P4-LbrHsp23–3’sgRNA (Table S1) and the *L. braziliensis* strain PER005 *HSP23* gene, explaining the lack of success for sgRNA set 4.

From the transfections with sgRNAs sets 1 and 2, three cell populations emerged: one with set 1 at day 18 post-transfection, and two with set 2, at day 18 and 25 post-transfection, respectively. From these three populations, clones were raised and expanded. Three clones were then subjected to whole genome sequencing: *HSP23*^–/–^ cl.1 and cl.2, from transfection with sgRNAs set 2; and *HSP23*^–/–^ cl.3, derived from the transfection with sgRNAs set 1. NGS analysis verified a lack of sequence reads for the targeted gene regions (Figure 2B), confirming site-specific disruption of the *LbrHSP23* ORF. Moreover, the precise integration of both drug-resistance cassettes in these *HSP23*^–/–^ mutant clones was also verified (Figure S6). Western blot analysis using specific antibodies [47] failed to detect HSP23 protein in the *HSP23*^–/–^ mutants (Figure 2C), confirming the null mutants on the genomic and proteomic levels. 

#### 3.2.2. *LbrHSP100* Gene Replacement 

**Figure 3:**
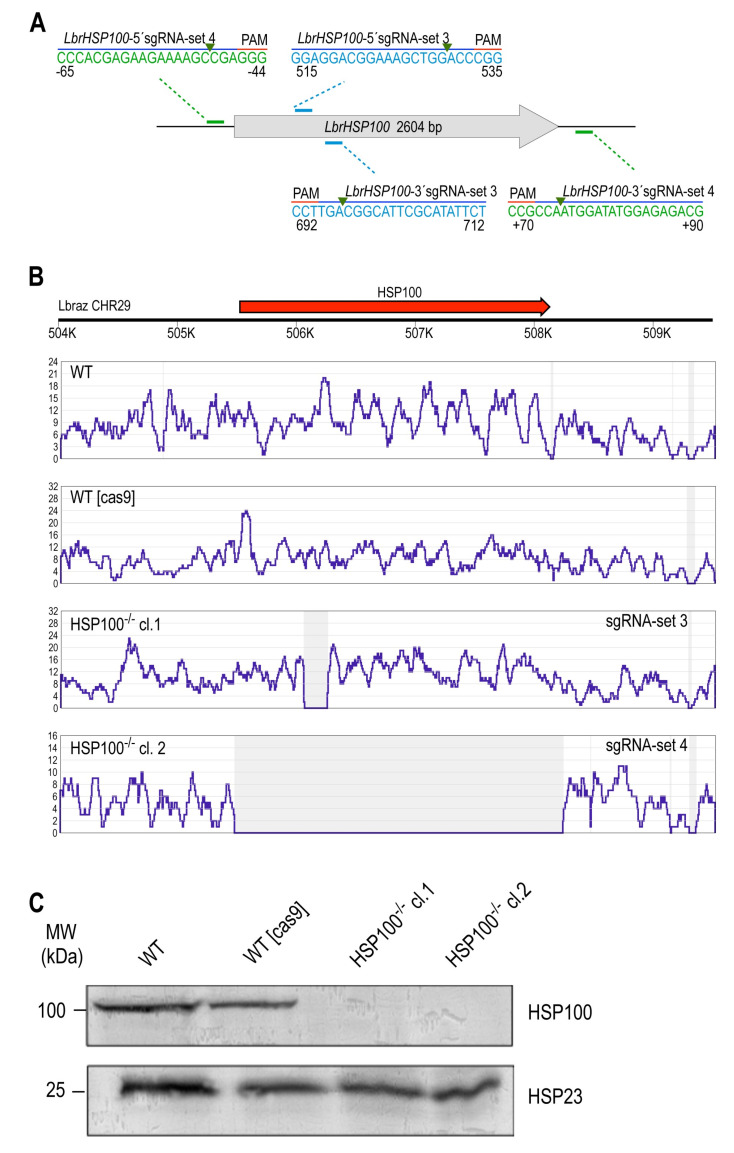
CRISPR–Cas9-mediated disruption of the endogenous *HSP100* gene in *L. braziliensis.* (**A**) For targeting *LbrHSP100* (LbrM.29.1350), two sets of sgRNAs tested (set 3 and set 4) worked efficiently. sgRNAs set 3 (*LbrHSP100*-513-5’sgRNA and *LbrHSP100*-712-3’sgRNA) targeted disruption of the *LbrHSP100* ORF in the N terminus. sgRNAs set 4 targeted 5’ and 3’ non-coding flanking sequences for *LbrHSP100* whole-gene deletion. Two cloned *L. braziliensis HSP100*^–/–^ lines were studied, *HSP100*^–/–^ cl.1 and *HSP100*^–/–^ cl.2, derived from transfection of set 3 or set 4 of *LbrHSP100*-targeting sgRNAs, respectively. (**B**) Whole genome sequencing of *HSP100*-null mutant lines. Sequence reads from each analysed strain were aligned to the reference DNA sequence consisting of chromosome 29 of *L. braziliensis* M2904 reference genome using Bowtie 2 software. The Y-axis represents the number of reads and the X-axis shows the nucleotide position (bp) on chromosome 29. Grey shaded areas denote complete lack of aligned reads. (**C**) Verification of *HSP100*-null mutants by Western blot analysis using anti-HSP100 (1/1000) antibody. Anti-HSP23 antibody (1/500) served as loading control. MW = Molecular weight in kilodalton.

sgRNA selection and replacement of the *LbrHSP100* gene were done following the same strategy. We obtained putative *LbrHSP100*-null mutants with sgRNAs set 3, targeting sequences in the N terminus of *LbrHSP100* ORF and set 4, targeting 5’ and 3’ non-coding sequences flanking the ORF for whole-gene deletion (Figure 3A). One cell population each emerged from the transfections and gave rise to multiple clones. Two *HSP100*^–/–^ clones obtained with sgRNAs sets 3 and 4, respectively, were then selected for further genetic and phenotypic characterisation. NGS analysis indeed confirmed the target-specific disruption of the *LbrHSP100* ORF and the on-target integration of both drug resistance cassettes at the predicted genomic sites for both *HSP100*^–/–^ mutants (Figure 3B; Figure S7). Western blot analysis using HSP100-specific antibodies [38] confirmed the lack of HSP100 in both mutants (Figure 3C).

To assess the fate of the Cas9/T7 construct (pTB007 episome) in the CRISPR-derived null mutants, we analysed Cas9 expression on the mRNA and protein levels by qRT-PCR and Western blot, respectively. Cas9 protein was undetectable in the three *HSP23*^–/–^ and two *HSP100*^–/–^ mutant clones (Figure S8A,B), alleviating concerns over phenotypic, off-target Cas9 effects.

### 3.3. L. braziliensis HSP23- and HSP100-Null Mutant Phenotypes Resemble Those Described for Old World Leishmania

For the phenotype analysis, we first attempted to create gene add-back parasites for both null mutants. In the *HSP23*^–/–^ mutants, we introduced the *LbrHSP23* transgene for integration into the 18S SSU rRNA locus, using the pIRmcs3+ vector [53], or as episome, using the over expression plasmid pCL1S-*LbrHSP23*. To generate the *HSP100* add-back cell lines, the *HSP100*^–/–^ mutants were transfected with the pIRmcs3+ vector harbouring *LbrHSP100* for genomic integration. Despite several attempts with different experimental conditions (data not shown), we could not generate any of the intended gene add-back cell lines. We suspect that the selection marker gene, coding for streptothricine N-acetyl transferase (SAT), was not stably expressed, possibly due to the known RNAi activity in *L. braziliensis* [9]. Ectopic gene expression from integrated and episomal transgenes is unpredictable in *L. braziliensis* (V.A., unpublished observations, and [59]).

**Figure 4:**
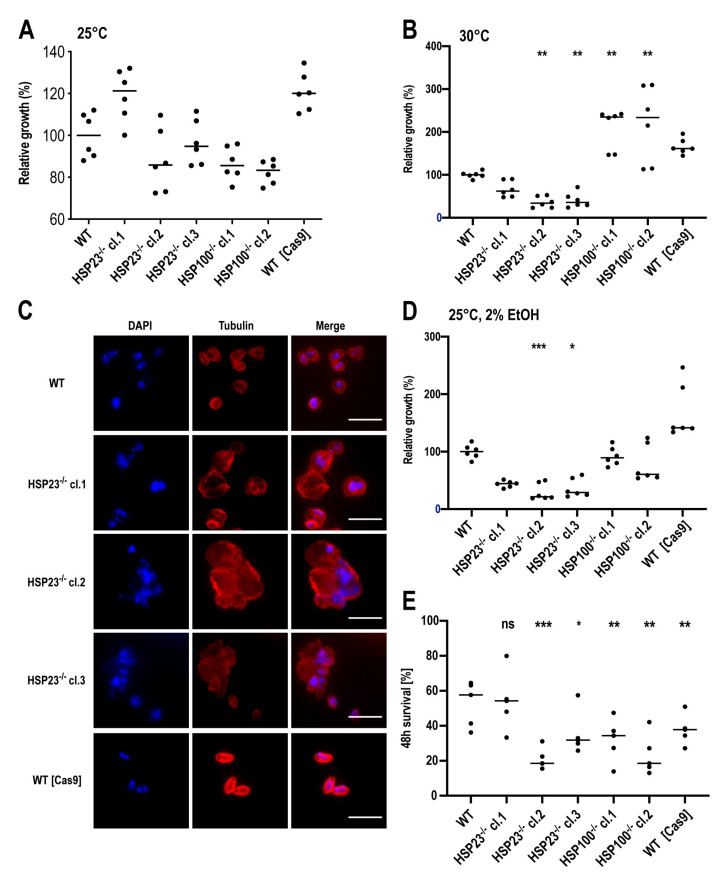
Phenotypic analyses of *L. braziliensis HSP23^–/–^* and *HSP100^–/–^* clones. For growth curves, promastigotes of WT, WT (Cas9), *HSP23*^–/–^ clones, and *HSP100*^–/–^ clones were seeded at a density of 1 × 10^6^ parasites/mL into 5 ml of complete M199 medium and grown for 4 days. Cell density was measured on day 4 and is shown as a percentage of WT cell density (set at 100%). Parasites were grown at 25 °C (**A**) and 30 °C (**B**). The *HSP23*^–/–^ clones incubated for 4 days at 30 °C were also stained with mouse anti-tubulin antibody (1/4000) and DAPI (1/50) (**C**). Images were taken on an EVOS FL Auto Cell Imaging System and processed using the ImageJ Software (https://fiji.sc). Scale bar: 10µm. Additional cultures were grown at 25 °C and pH 7.4 with the addition of 2% ethanol (**D**). The horizontal black lines in panels A, B, and D indicate the median of 6 biological samples from 3 separate experiments. Significance was tested using the Kruskal–Wallis test; * *p* < 0.05, ** *p* < 0.01, *** *p* < 0.001. (**E**) Primary mouse bone-marrow-derived macrophages were differentiated and infected with stationary-phase promastigotes of WT, WT [Cas9], *HSP23*^–/–^ clones, and *HSP100*^–/–^ clones at a MOI of 1:8 (macrophage-to-parasite ratio). After 4 h, free parasites were washed away and the infected macrophage cultures were further incubated at 34 °C under 5% CO_2_ for 44 h. Genomic DNA from *Leishmania*-infected macrophages was isolated at 4.5 h and at 48 h post-infection, and parasite load was determined by TaqMan qPCR quantifying parasite *actin* gene DNA relative to host macrophage *actin* gene DNA. Shown is intracellular parasite survival [%] after 48 h, with the bar indicating the median of *n* = 5. Ratio-paired, one-sided Student’s *t*-test: * *p* < 0.05, ** *p* < 0.01, *** *p* < 0.001 between data pairs. ns = not significant.

We nevertheless proceeded to test the growth phenotypes of the *L. braziliensis HSP23*^–/–^ and *HSP100*^–/–^ null mutants under various *in vitro* growth conditions compared with the wild-type and with Cas9-expressing cells. Cell density on day 4 (stationary phase) was analysed and displayed as percentage of growth relative to the wild type (set at 100%). Under optimal *in vitro* growth conditions for promastigotes (25 °C, pH 7.4), the *L. braziliensis* PER005cl2 wild-type strain achieved a median 24.9-fold growth (2.49 × 10^7^ cells/ml). Two *HSP23*^–/–^ null mutants, *HSP23*^–/–^ cl.2 and *HSP23*^–/–^ cl.3, grew at rates similar to the wild type (median relative growth: 85.0% for *HSP23*^–/–^ cl.2 and 93.4% for *HSP23*^–/–^ cl.3; Fig. 4A). *HSP23*^–/–^ cl.1 displayed a 20% elevated proliferation, similar to the Cas9-expressing cells. The *HSP100*-null mutants showed proliferation rates (median relative growth: 86.1% for *HSP100*^–/–^ cl.1 and 81.8% for *HSP100*^–/–^ cl.2) comparable to those of the wild type (Figure 4A). Therefore, we see no growth phenotype for *HSP23*^–/–^ and *HSP100*^–/–^ null mutants under optimal culture conditions. This is in keeping with earlier findings about the significance of HSP100 and HSP23 in the promastigote [47,56].

.

Stable Cas9 expression from the pTB007 episome increased the growth rate of *L. braziliensis* promastigotes at 25 °C (Figure S1C), leading to a higher cell density in late-log phase (day 3; *p* = 0.004, *U* test) and in stationary phase (day 4; *p* = 0.015, *U* test) compared to the wild-type parasites, likely reflecting a positive effect on cell proliferation, similar to previous observations [21].

Next, we repeated the analysis at 30 °C, the upper temperature limit for *L. braziliensis* growth *in vitro* [60]. Proliferation of the *L. braziliensis* PER005cl2 wild-type strain was slowed considerably at 30 °C, reaching a median of 4.9 × 10^6^ cells/ml at day 4 (4.9-fold growth). The *L. braziliensis HSP23^–/–^* null mutants, particularly *HSP23*^–/–^ cl.2 and *HSP23*^–/–^ cl.3, were sensitive to the 30 °C cultivation temperature and did not proliferate (Figure 4B). This temperature-sensitive phenotype is in line with previous work with *L. donovani HSP23^–/–^* null mutants [47]. We also tested the cell integrity of the *L. braziliensis HSP23*^–/–^ null mutants at 30 °C. As shown by immunofluorescence microscopy (Figure 4C), all three *L. braziliensis HSP23*-null mutants showed abnormally rounded, swollen and irregular shapes, and formed cell aggregates indicating cellular damage. These changes were not observed in the control cells, *L. braziliensis* wild type and Cas9-expressing cells, which presented as individual, well defined cells. 

Conversely, the *L. braziliensis HSP100*^–/–^ null mutants were fully viable and proliferating at 30 °C, even exhibiting a significant growth advantage over the wild type (Figure 4B). This temperature tolerance of the *L. braziliensis HSP100*^–/–^ null mutants matches previous findings from phenotype analyses of *L. donovani HSP100*^–/–^ null mutants [57], but contrasts with the phenotype of *L. major HSP100*^–/–^ null mutants, which were hypersensitive at the upper limit of growth temperature [56]. Lastly, the Cas9-expressing cells grown at 30 °C also showed an elevated growth without reaching statistical significance (Figure 4B). 

We next tested the *L. braziliensis HSP23*^–/–^ and *HSP100*^–/–^ null mutants for tolerance to sublethal ethanol concentrations, a trigger of the unfolded protein response, a stress signalling pathway of the endoplasmic reticulum (ER) that is related to the heat shock response [61,62]. Treatment with 2% ethanol caused growth reduction for all three *L. braziliensis HSP23*^–/–^ null mutants (Figure 4D). This increased sensitivity of *L. braziliensis HSP23*^–/–^ null mutants to a chemical stressor (i.e., ER stress-sensitive phenotype) is in agreement with previous work in *L. donovani HSP23*^–/–^ mutants [47], further supporting the involvement of HSP23 in protecting *Leishmania* against protein misfolding stress. The *HSP100*^–/–^ null mutants were not affected by exposure to 2% ethanol (Figure 4D). Again, the Cas9-expressing cells showed a slightly increased growth compared to the wild type (Figure 4D). 

Lastly, we tested the ability of the wild type and mutant strains to survive inside macrophages. Primary mouse bone marrow-derived macrophages were differentiated and infected *in vitro* at a parasite to macrophage ratio of 8:1 using stationary-phase promastigotes. The parasite load was evaluated by qPCR [50] at 48 h post infection relative to the parasite load after 4.5 h of parasite internalisation. 

The average percentage of surviving *L. braziliensis* PER005cl2 wild-type parasites within macrophages at 48 h post-infection was 52.6 ± 13.0% (Figure 4E). The loss of HSP100 had a significant impact on the intracellular survival of the two *L. braziliensis HSP100* null mutants. The effect was more pronounced for the whole-gene deletion mutant (*HSP100*^–/–^ cl.2; mean survival ± SD: 23.4 ± 11.7%) than for the partial gene disruption (*HSP100*^–/–^ cl.1; 32.0 ± 12.4%) (Figure 4E). The impaired ability of these *L. braziliensis HSP100*^–/–^ null mutants for intracellular survival in *in vitro*-infected mouse macrophages was also documented for *L. major* and *L. donovani HSP100*-null mutants [56,57].

The ability to survive in macrophages was affected in only two *L. braziliensis HSP23*^–/–^ mutants (*HSP23*^–/–^ cl.2: mean survival ± SD: 21.2 ± 6.1%; *HSP23*^–/–^ cl.3: 35.6 ± 12.5%)(Figure 4E), whereas the *HSP23*^–/–^ cl.1 was able to survive intracellularly (54.1 ± 16.8%) at a rate similar to the wild-type parasites (Figure 4E). The reduced survival of *HSP23*^–/–^ cl.2 and cl.3 matches the poor growth of these clones at elevated temperature and under ethanol stress (Figure 4B,D) and is in line with previous work performed with a *L. donovani HSP23*-null mutant [47].

In a first attempt to investigate possible genomic adaptations in the mutants as cause for varying phenotypes, we evaluated aneuploidy patterns. Using the NGS sequence reads from the WGS analysis and quantifying normalised sequence read densities for individual chromosomes in *L. braziliensis* WT cells, WT [Cas9] cells, three *HSP23*^–/–^ mutant clones and two *HSP100*^–/–^ mutant clones, we calculated chromosome ploidies (Figure S9A). Indeed, we found profound differences between *L. braziliensis HSP23*^–/–^ mutants themselves and compared to the other parasite strains. *HSP23*^–/–^ clone 1 is trisomic for chromosome 30 and shows intermediate somy (2.56) for chromosome 4. *HSP23*^–/–^ clone 2 shows a marked increase of chromosome 2 ploidy (4.82). *HSP23*^–/–^ clone 3 shows strong amplification (4.6) of chromosome 14, trisomies for chromosomes 18, 33 and 34, and a slight (2.39) increase for chromosome 4, which was also partly amplified in *HSP23*^–/–^ clone 1. The strong increase of chromosome 2 sequence reads for *HSP23*^–/–^ clone 2 is due to an apparent amplification of a ~20,000 bp region between positions 260,000 and 280,000 (Figure S9C). The amplified region contains mostly copies of a SLACS retrotransposon (LbrM.02.0550), and a possible context with the loss of HSP23 is not obvious. 

All three *L. braziliensis HSP23*^–/–^ clones, but also the Cas9-expressing strain were trisomic for chromosome 26, possibly causing the minor fitness gain observed for the Cas9 strain.

### 3.4. Complementation Studies in L. major HSP23-Null Mutants Indicate a Conserved Function in Thermotolerance for Trypanosomatid HSP23

**Figure 5:**
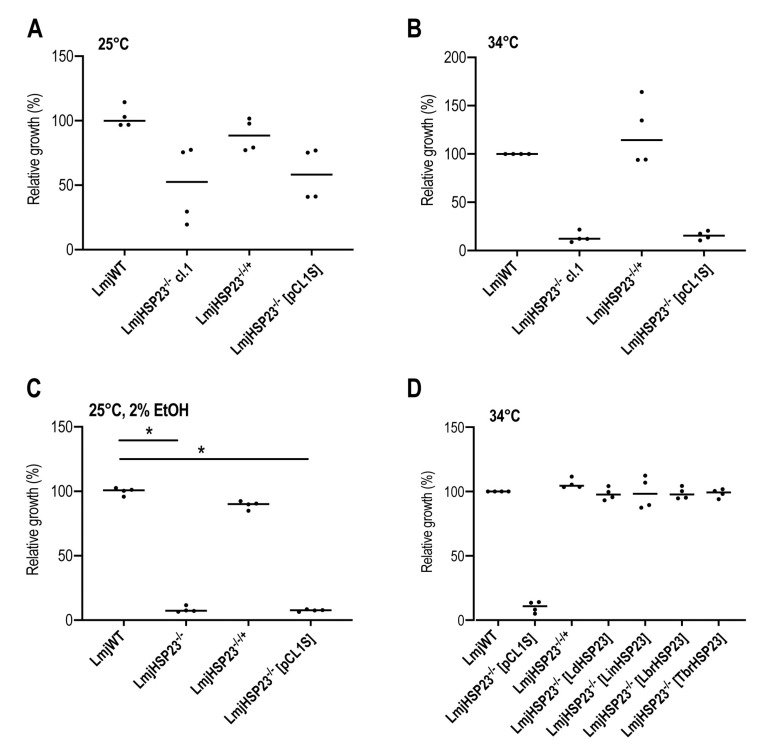
Phenotypic analysis of *L. major* *HSP23^–/–^* mutants and complementation strains. 1 × 10^6^ or 5 × 10^6^ parasites/ml were seeded in 10 ml complete M199 medium and parasite density was assessed at day 4. Parasites were grown at 25 °C (**A**), 34 °C (**B**), and 25 °C with 2% EtOH (**C**). Cell density is shown as percentage of WT (set at 100%). (**D**) Complementation studies in *LmjHSP23*^–/–^ mutants. Null mutants were transfected with the pCL1S over expression vector harbouring the *HSP23* gene of *L. major, L. donovani, L. infantum, L. braziliensis*, and *Trypanosoma brucei* or with the empty vector only. Complementation populations were subjected to growth experiments at 34 °C. Cell density was assessed at day 4 and is shown normalised to *Lmj* WT growth (set at 100%). * = *p* < 0.05.

The failure to establish ectopic HSP23 expression in the *L. braziliensis HSP23*^–/–^ clones precluded a conclusive correlation between loss of HSP23 and the observed phenotypes. To complement this, we also produced CRISPR-derived *L. major HSP23*^–/–^ null mutants, following the same experimental strategies. Three selected *L. major HSP23*^–/–^ null mutant clones (*LmjHSP23*^–/–^ cl.1–cl.3) were analysed by whole genome sequencing, confirming the successful replacement of the *LmjHSP23* gene (Figure S10A) and the correct integration of both drug-resistance cassettes (Figure S11). Further verification by Western blot analysis using HSP23-specific antibodies showed a lack of the HSP23 protein in all *L. major HSP23*^–/–^ null mutants (Figure S10B). From these clones, we selected *LmjHSP23*^–/–^ cl.1 for genetic complementation and phenotypic analyses. We introduced the *LmjHSP23* transgene as episome to generate a *LmjHSP23* add-back cell line. In vitro, at optimal growth conditions for promastigotes (25 °C, pH 7.4), the null mutant showed a 50% reduced growth compared with wild-type cells (Figure 5A). This reduced growth of the null mutant could be restored to near-wild type levels by the *LmjHSP23* transgene, but not by the empty expression plasmid pCL1S (Figure 5A). At 34 °C, a temperature relevant for dermotropic *Leishmania* species, the *LmjHSP23*^–/–^ cl.1 mutant promastigotes were severely affected and did not proliferate (Figure 5B). This temperature-sensitive phenotype was rescued by the *LmjHSP23* transgene (Figure 5B), similar to what was reported for *L. donovani HSP23*^–/–^ mutants [47]. We also tested the *LmjHSP23*^–/–^ cl.1 mutant for tolerance to sublethal ethanol stress. A 2% ethanol exposure caused growth inhibition in the null mutant (Figure 5C), but not in the *LmjHSP23^–/–^* (LmjHSP23) parasites (Figure 5C). Thus, we established *LmjHSP23*^–/–^ cl.1 as a suitable host strain for the functional complementation with various trypanosomatid *HSP23* genes.

A similar ploidy analysis was also performed for *L. major* WT, *L. major* WT [Cas9] and the two *L. major HSP23^–/–^* clones, 1 and 2 (Figure S9B). Except for a very minor increase for chromosomes 5, 6, and 8, no karyotypic changes could be observed.

The *LmjHSP23*^–/–^ cl.1 mutant was transfected with pCL1S bearing the *L. donovani, L. infantum*, *L. major*, *L. braziliensis*, or *T. brucei HSP23* orthologs, respectively. Ectopic expression of these transgenes was verified at the RNA level using qRT-PCR analysis with *HSP23* species-specific primers, showing varying rates of over expression (Figure S12A). We also verified the HSP23 protein level by Western blot analysis using specific antibodies raised against *L. donovani* HSP23 [47]. Over expression was confirmed for all *Leishmania* HSP23 homologs, except for the putative *T. brucei* HSP23 (Figure S12B), the latter likely due to low amino acid sequence conservation (36%) between the *L. donovani* and *T. brucei* HSP23 homologs.

We then tested whether the temperature-sensitive phenotype of the *L. major HSP23^–/–^* mutant could be complemented by the HSP23-encoding, orthologous genes from other *Leishmania* species and the closely related *Trypanosoma brucei*. These supposed HSP23 homologs share between 36% and 99% amino acid sequence identity (Table S2). At 34 °C, all trypanosomatid *HSP23* transgenes restored growth of *L. major HSP23*-null mutants to wild-type levels, abrogating the mutant phenotype (Figure 5D). This shows that all trypanosomatid HSP23 homologs share the same functionality, conferring protection against heat stress, and likely maintaining protein folding homeostasis in trypanosomatid organisms. Furthermore, the functional conservation of HSP23 homologs among the Trypanosomatidae confirms the phenotypes we observed in the *L. braziliensis HSP23*-null mutants, since LbrHSP23 expression can restore thermotolerance to the *L. major HSP23*^–/–^ mutant.

## 4. Discussion

The protozoan parasite *Leishmania braziliensis* is one of the most pathogenic dermotropic *Leishmania* species circulating in the Americas, where it is the main cause of cutaneous and mucocutaneous leishmaniasis [4,63]. Despite its prevalence and importance to public health, *L. braziliensis* has been less studied and is therefore less experimentally developed compared to Old World *Leishmania* species such as *L. major* and *L. donovani*, which have been traditionally used as models for studying the biology of these obligate intracellular parasites. Given that *L. braziliensis* is a member of the subgenus *Viannia,* with a considerable phylogenetic distance to the Old World species and even to the Central and South American *L. mexicana* complex, conservation of gene function between the subgenera may not be assumed automatically, and may require experimental confirmation by reverse genetics. 

One of the main approaches for genetic modification of *Leishmania* parasites to probe gene function has been the generation of gene replacement mutants by homologous recombination-mediated replacement [5,64], which allows the creation of null mutants and their subsequent phenotypic analysis [6,65]. While this has proven a powerful genetic tool in Old World *Leishmania* spp., but also in Central American *L. mexicana* [66], our literature search did not turn up any work regarding homologous recombination-based gene replacement in *L. braziliensis*. Studies reporting on the use of homologous recombination in *L. braziliensis* demonstrate the generation of stable transgenic parasite lines from integration of DNA constructs into the SSU rDNA genomic locus. These include *L. braziliensis* lines expressing reporter genes, e.g., luciferase or eGFP, which hold potential for parasite tracking and monitoring effects of antileishmanial compounds *in vitro* and *in vivo* [67,68,69], and over expressing parasite lines for the analysis of gene products, e.g., to assess antimony susceptibility and resistance mechanisms [70,71,72]. Moreover, circular extrachromosomal cosmids can be stably introduced into *L. braziliensis* to over-express stretches of genomic DNA and connect the over expression phenotypes to biological processes such as virulence [73] and antimony resistance [59]. The experimental proof that *L. braziliensis* is a RNAi-competent species started the development of RNAi-based gene knockdown strategies for the loss-of-function phenotyping of genes in this species [9,10]. More recently, the CRISPR–Cas9 technology, with its advantages of being less time-consuming than traditional gene targeting and less susceptible to off-target effects than RNAi-based approaches [74], has added to the genetic toolbox that is available for the study of *Leishmania* spp. [19,20], allowing researchers to investigate gene functions with unprecedented ease, accuracy, efficiency, and scale in biological contexts [17,25,29,40]. 

In this study, we report the application of CRISPR–Cas9-mediated gene editing to the efficient and precise disruption of two endogenous, non-essential, single-copy genes and one integrated transgene in *L. braziliensis*. We opted for a CRISPR–Cas9, molecular cloning-free method developed for the use in *Leishmania* that relies on T7 RNAP-based expression of sgRNAs in vivo [17]. For this, we first generated a parental *L. braziliensis* cell line expressing Cas9 and T7 RNAP. Since plasmid pTB007 was designed for integration of both transgenes into the *L. major* beta-tubulin locus [17], we transfected pTB007 as stable, circular episome under hygromycin B selection. This episome was well tolerated by *L. braziliensis* strain PER005cl2 used in this study and was stably maintained for several months, with no apparent Cas9 toxicity during in vitro promastigote passage, indicating that this episomal transgene could be maintained without inducing deleterious RNAi effects in *L. braziliensis*. 

For our study, we used a cloned *L. braziliensis* strain, derived from a clinical isolate, whose entire genome had been sequenced [46].This allowed us to select correct, highly specific sgRNA templates and donor DNAs for precise, targeted gene editing with no predicted off-target mutations. The original clinical isolate from which PER005cl2 strain is derived, was shown to be infective for primary mouse peritoneal macrophages [34], within which it is sensitive to pentavalent antimony. Furthermore, this isolate was confirmed not to harbour *Leishmaniavirus* LRV1 [75], a cytoplasmic double-stranded RNA virus frequently found as endosymbiont in *Leishmania* (*Viannia*) species [75,76,77], and which appears to enhance virulence and persistence of its *Leishmania* host [78,79]. 

We first targeted an eGFP coding sequence inserted into the SSU rRNA coding gene(s) of the *L. braziliensis* parental Cas9/T7 cell line. We applied double antibiotic selection after CRISPR targeting, using increasing antibiotic pressure at two time points, i.e., predetermined minimal effective concentrations of antibiotics at 24 h post-transfection and until transfectants emerged in culture, followed by higher antibiotic selection pressure to enrich for homozygously edited cells, and found this to be an effective strategy. The *eGFP* editing in *L. braziliensis* was assessed at the cell population level and compared to that achieved in *L. donovani*. Overall, we observed a different activity for the same pairs of sgRNAs in the two *Leishmania* species studied. While all 6 sgRNA sets that targeted sites within the *eGFP* gene were highly active in *L. donovani*, they had a wide range of efficiency in *L. braziliensis*. The most active sgRNAs (sets 5 and 6) were the same in *L. braziliensis* and *L. donovani*, indicating that the sgRNA sequence had an impact on the gene targeting efficiency. This is in line with a recent study that tested the efficiency of three gRNAs targeting identical sequences of the miltefosine transporter gene in *L. donovani*, *L. major*, and *L. mexicana*, and found the relative gRNA activity to be the same [31]. Studies in other systems revealed that sgRNA sequence features such as position-specific nucleotide composition, GC content, motifs located in the sgRNA “seed” region, and secondary structures of sgRNAs contribute to sgRNA efficacy [80,81,82,83,84].

The different gene targeting efficiencies of the same sgRNA sets observed for *L. braziliensis* and *L. donovani* may be due to different factors. First, the presence of an active RNAi machinery in *L. braziliensis* [9] may have an effect on ectopic Cas9 and T7 RNAP expression from episomal DNA constructs in this species, as was shown before [59]. Second, there may be differences in the T7-dependent expression level of different sgRNAs and Cas9 among *Leishmania* species [31]. We have used T7 RNAP-driven in vivo expression of sgRNA templates that were delivered to the *Leishmania* parental Cas9/T7 cell lines by transient transfection [17]. Variation of T7 RNAP-mediated transcription may lead to different intracellular levels of sgRNA that may limit the efficiency of Cas9-dependent DNA cleavage. A recent study suggested that a threshold level for both Cas9 and sgRNA expression is required for an efficient CRISPR-mediated gene knockout, which in turn is determined by the specific potency of a given sgRNA [85]. In keeping with this, increased sgRNA expression and maturation dramatically improved the efficiency of CRISPR–Cas9 mutagenesis in *Candida albicans* [86]. Thirdly, DSB repair efficiency may differ between *Leishmania* species [31]. Fourth, small variations in the intrinsic antibiotic sensitivity of different *Leishmania* species and strains may cause differences in transgene copy numbers, both for the integrated *GFP* gene and for the Cas9/T7-RNAP construct, leading to different efficiencies. Lastly, other factors playing a role in the biology of the *Leishmania* species studied may also play a role, such as variations of chromatin structure. 

In our experiments, the copy numbers of *eGFP* within the SSU rRNA gene units of the *L. braziliensis* Cas9/T7/eGFP parental cell line were not determined. Assuming one copy of *eGFP* present per genome in the *L. braziliensis* Cas9/T7/eGFP, as shown in a recent study focused on the same species [87], our results suggest that the *eGFP*-specific sgRNA sets 1, 2, 3, and 4 generated mono-allelic edits, i.e., single-allele replacements, whereas the most efficient sgRNAs, sets 5 and 6, generated mostly double-allelic edits.

We were also able to efficiently disrupt two non-essential, endogenous, single-copy genes of *L. braziliensis* encoding the heat shock proteins HSP23 and HSP100. We obtained double-allelic, Cas9-free *HSP23*^–/–^ and *HSP100*^–/–^ null mutants. The in vitro phenotypes of the *L. braziliensis HSP23*- and *HSP100*-null mutants were assessed and compared to the wild-type strain, since gene add-back variants could not be obtained. Nevertheless, the analysis of independently cloned mutant cell lines revealed largely consistent phenotypes, strengthening the correlation between the disruption of the target gene and the loss-of-function phenotypes. This was further supported by the complementation studies carried out in the *L. major HSP23*-null mutant, which demonstrated functional homology between the *HSP23* genes of the Trypanosomatidae. Furthermore, the rapid loss of the Cas9 episome in the absence of antibiotic selection is important when evaluating the phenotype, as the WT [Cas9] strain which was kept under selection showed a divergent phenotype from the wild type. We would therefore refrain from using genomic integration constructs for the expression of Cas9.

We do not know the reason behind the different capacity of intracellular amastigotes from the three studied *L. braziliensis HSP23*^–/–^ mutants to survive inside macrophages. All parasite strains/clones were subjected to the same *in vitro* culture, electroporation, cloning, antibiotic selection, and stress conditions. They had similar passage numbers before phenotype analyses, and their phenotypes were investigated in parallel in all assays. Moreover, the CRISPR–Cas9 components were no longer present when single-cell cloning was performed. We suspected that the mutant clones might have undergone some level of genetic adaptation, e.g., via spontaneous mosaic aneuploidy followed by selection for vitality. We observed a similar, spontaneous loss of phenotype for a *L. donovani HSP23*^–/–^ clone, due to amplification of the gene coding for casein kinase 1.2 [45]. We indeed found ploidy changes that were specific to the *L. braziliensis HSP23*^–/–^ mutants. One of those, a trisomy of chromosome 34, which harbours the casein kinase 1.2 gene in *L. braziliensis*, may have a similar effect as in *L. donovani*. 

Lastly, an average of 37.7% (± 8.6%) *L. braziliensis* Cas9-expressing cells were able to survive inside macrophages (Figure 4E). Those cells show a trisomy for chromosome 26, similar to all three *L. braziliensis HSP23*^–/–^ clones (Figure S9A). This trisomy is absent from the wild type and from the two *L. braziliensis HSP100*^–/–^ clones.

## 5. Conclusions

*Leishmania (V.) braziliensis* is amenable to reverse genetics using a CRISPR–Cas9 protocol as shown in this work. Gene replacement occurs exclusively at the predicted sites. As is known, ectopic expression of the genes of interest presents a problem, due to the effects of RNAi in the *Viannia* subgenus. The functions of at least two amastigote-specific heat shock proteins, HSP100 and HSP23, are conserved between Old World and New World leishmaniae and likely in *T. brucei* as well. With a workable protocol for gene replacement now in place, urgent questions pertaining to the biology of the *Viannia* subgenus can now be addressed by means of reverse genetics.

## Supplementary Materials

The following are available online at https://www.mdpi.com/2073-4425/11/10/1159/s1, Figure S1: Detection of Cas9 and T7 RNAP in the *L. braziliensis* parental cell line; Figure S2: Flow cytometric analysis results for *L. donovani* eGFP-Cas9-expressing promastigotes transfected with *eGFP*-targeting sgRNAs; Figure S3: Flow cytometric analysis results for *L. braziliensis* eGFP-Cas9-expressing promastigotes transfected with *eGFP*-targeting sgRNAs; Figure S4: PCR verification of *eGFP* gene replacement; Figure S5: Titration of selection antibiotics on *L. braziliensis* promastigotes; Figure S6: Verification of the *L. braziliensis HSP23* gene replacement by the respective resistance cassettes; Figure S7: Verification of the *L. braziliensis HSP100* gene replacement by the respective resistance cassettes; Figure S8: Cas9 expression in *L. braziliensis HSP23*- and *HSP100*-null mutants; Figure S9: Karyotype analysis of *L. braziliensis* and *L. major* strains; Figure S10: Verification of *L. major HSP23*-null mutants; Figure S11: Verification of replacement cassette integration into the *L.major HSP23* locus; Figure S12: Verification of *L.major HSP23*^-/-^ complementation lines; Table S1: List of primers used in this study; Table S2: Sequence identity analysis of trypanosomatid HSP23 proteins.
