# Supplementary material for: Application of CRISPR/Cas9-Based Reverse Genetics in Leishmania braziliensis: Conserved Roles for HSP100 and HSP23
Source: Genes (Basel). 2020 Sep 30;11(10):1159. doi: 10.3390/genes11101159 (PMC7601497; doi:10.3390/genes11101159)
Supplement: Supplementary file 1 [file genes-11-01159-s001.pdf]

Application of CRISPR/Cas9-Based Reverse Genetics in *Leishmania braziliensis*: Conserved Roles for HSP100 and HSP23

Vanessa Adaui [1,2,5,†], Constanze Kröber–Boncardo [1,†], Christine Brinker [1], Henner Zirpel [1,6], Julie Sellau [1], Jorge Arévalo [2], Jean–Claude Dujardin [3,4] and Joachim Clos [1,*]

[1] Bernhard Nocht Institute for Tropical Medicine, Hamburg D-20359, Germany; vanessa.adaui@upc.edu.pe (V.A.), kroeber@bnitm.de (C.–K.B.), brinker@bnitm.de (C.B.), henner–444@gmx.net (H.Z.), sellau@bnitm.de (J.S.)
[2] Instituto de Medicina Tropical Alexander von Humboldt, Universidad Peruana Cayetano Heredia, Lima 15102, Peru; jorge.arevalo@upch.pe
[3] Institute of Tropical Medicine, Antwerp 2000, Belgium; JCDujardin@itg.be
[4] Department of Biomedical Sciences, University of Antwerp, Antwerp 2000, Belgium

Current affiliations:
[5] Centre for Research and Innovation, Faculty of Health Sciences, Universidad Peruana de Ciencias Aplicadas, Lima 15067, Peru
[6] City of Hope National Medical Center, Duarte 91010, CA, USA
† These authors contributed equally to this work.
* Corresponding author: clos@bnitm.de; Tel.: +49-40-42818481

Received: 04 September 2020; Accepted: 25 September 2020;

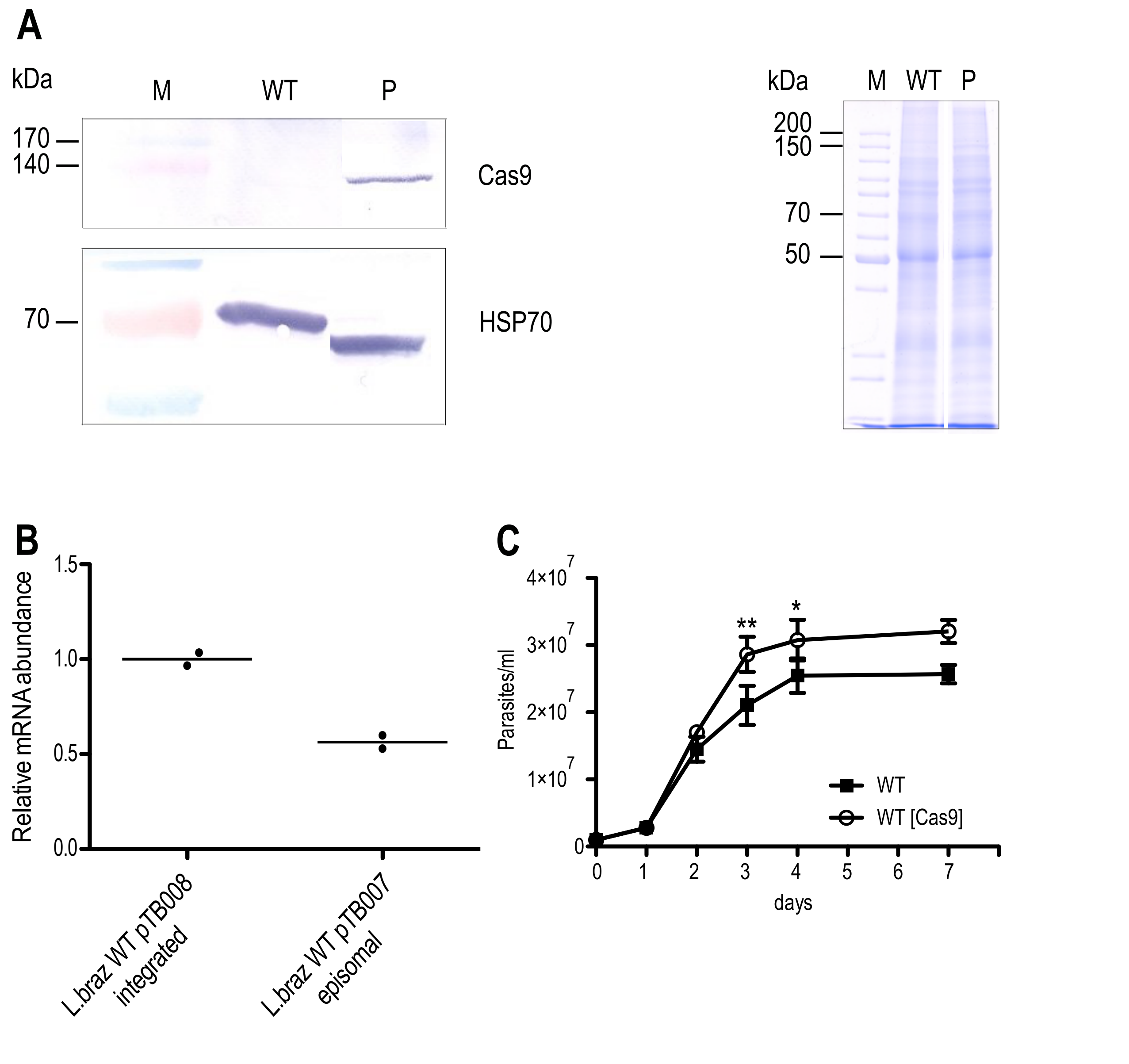

Figure S1. Detection of Cas9 and T7 RNAP in the *L. braziliensis* parental cell line.
(A) Western blot of whole cell lysates of *L. braziliensis* PER005cl2 wild type (WT) and *L. braz:*Cas9 (Cas9 T7) parental cell line (P) probed with anti-FLAG (1/500) or anti-HSP70 (1/500; loading control). M, protein molecular weight marker. (B) Relative mRNA abundance of *T7 RNAP* quantified by qRT-PCR. Samples: *left*, *L.braz* WT strain transfected with the linearised pTB008 plasmid (encoding T7 RNAP) for integration into the SSU rRNA locus, used as calibrator sample; *right*, parental cell line *L. braz:*Cas9 (transfected with the pTB007 episome). (C) Impact of stable Cas9 expression on the growth of *L. braziliensis* promastigotes. Promastigotes of *L.braz HSP23*$^{+/+}$ WT and WT [Cas9] were seeded at a density of 1×10^6 parasites/ml into 5 ml of complete M199 medium (in 25-cm^2 cell culture flasks) and cultured at 25°C for 7 days. The cell density was measured on days 1, 2, 3, 4 and 7 in two biological replicates in three independent experiments. The data represent the mean +/- SD. **P*<0.05, ***P*<0.01 (Mann-Whitney test).

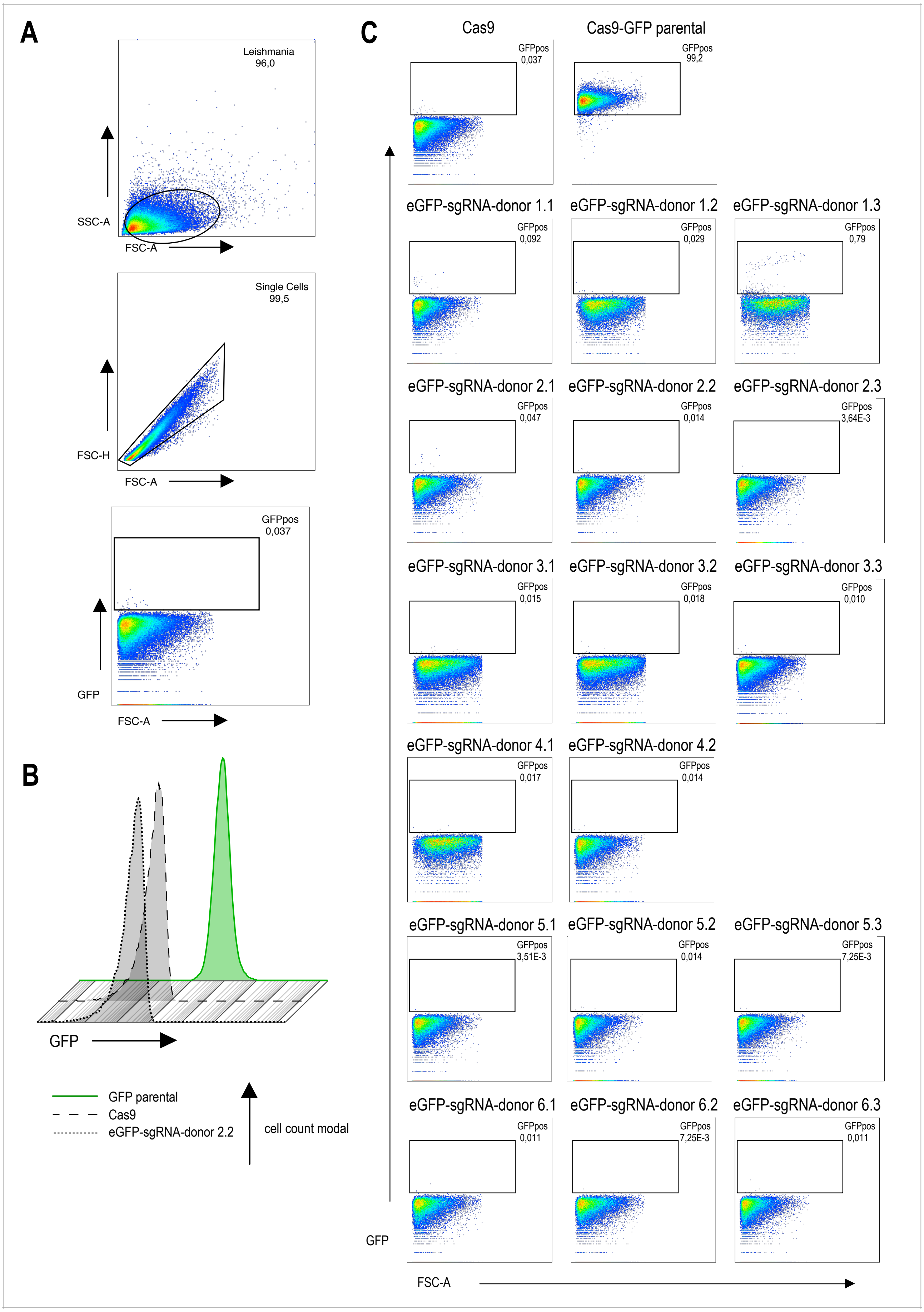
A
Leishmania
96,0
SSC-A
FSC-A
Single Cells
99,5
FSC-H
FSC-A
GFPpos
0,037
GFP
FSC-A
B
GFP
GFP parental
Cas9
eGFP-sgRNA-donor 2.2
cell count modal
C
Cas9
GFPpos
0,037
Cas9-GFP parental
GFPpos
99,2
eGFP-sgRNA-donor 1.1
GFPpos
0,092
eGFP-sgRNA-donor 1.2
GFPpos
0,029
eGFP-sgRNA-donor 1.3
GFPpos
0,79
eGFP-sgRNA-donor 2.1
GFPpos
0,047
eGFP-sgRNA-donor 2.2
GFPpos
0,014
eGFP-sgRNA-donor 2.3
GFPpos
3,64E-3
eGFP-sgRNA-donor 3.1
GFPpos
0,015
eGFP-sgRNA-donor 3.2
GFPpos
0,018
eGFP-sgRNA-donor 3.3
GFPpos
0,010
eGFP-sgRNA-donor 4.1
GFPpos
0,017
eGFP-sgRNA-donor 4.2
GFPpos
0,014
eGFP-sgRNA-donor 5.1
GFPpos
3,51E-3
eGFP-sgRNA-donor 5.2
GFPpos
0,014
eGFP-sgRNA-donor 5.3
GFPpos
7,25E-3
eGFP-sgRNA-donor 6.1
GFPpos
0,011
eGFP-sgRNA-donor 6.2
GFPpos
7,25E-3
eGFP-sgRNA-donor 6.3
GFPpos
0,011
GFP
FSC-A

Figure S2. Flow cytometric analysis results for *L. donovani* eGFP-Cas9-expressing promastigotes transfected with *eGFP*-targeting sgRNAs.
(A) Gating scheme used to identify the proportion of *L. donovani* promastigotes that were GFP-positive.
(B) Representative histogram of GFP expression in the Cas9-eGFP-expressing parental cell line (positive control) compared to an *eGFP*-null mutant (Cas9-derived KO) and Cas9-expressing (GFP-negative) parasites.
(C) Plots showing the percentage of GFP-positive parasites following Cas9-mediated *eGFP* disruption. Six different sets of sgRNAs co-transfected with donor DNAs were tested in triplicate, except for set 4 (*eGFP*-sgRNA-donor 4) that was only tested in duplicate. Pairs of sgRNAs tested are described in the legend of Fig. 1B.

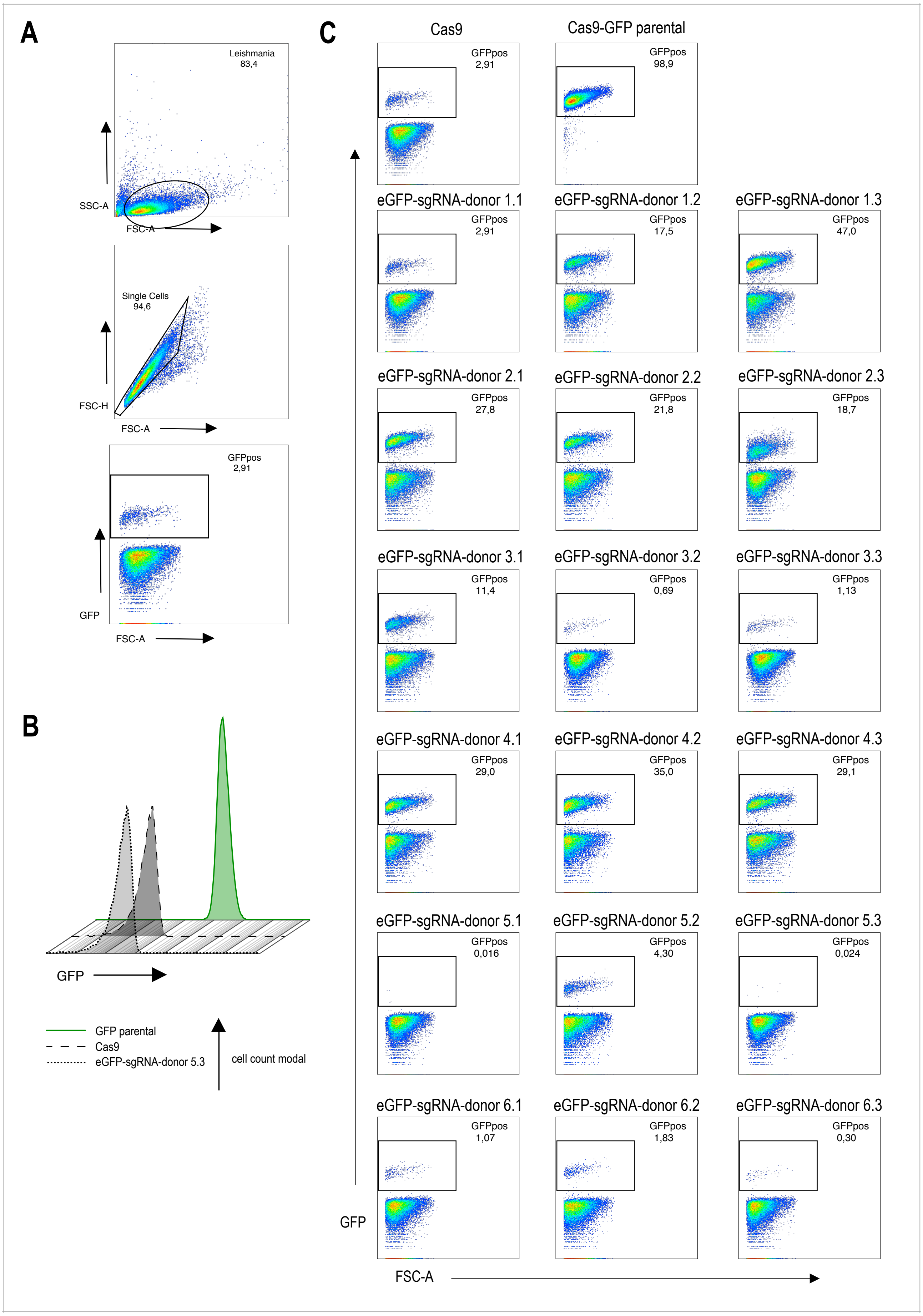

A
Leishmania
83,4
SSC-A
FSC-A
Single Cells
94,6
FSC-H
FSC-A
GFPpos
2,91
GFP
FSC-A
B
GFP
GFP parental
Cas9
eGFP-sgRNA-donor 5.3
cell count modal
C
Cas9
GFPpos 2,91
Cas9-GFP parental
GFPpos 98,9
eGFP-sgRNA-donor 1.1
GFPpos 2,91
eGFP-sgRNA-donor 1.2
GFPpos 17,5
eGFP-sgRNA-donor 1.3
GFPpos 47,0
eGFP-sgRNA-donor 2.1
GFPpos 27,8
eGFP-sgRNA-donor 2.2
GFPpos 21,8
eGFP-sgRNA-donor 2.3
GFPpos 18,7
eGFP-sgRNA-donor 3.1
GFPpos 11,4
eGFP-sgRNA-donor 3.2
GFPpos 0,69
eGFP-sgRNA-donor 3.3
GFPpos 1,13
eGFP-sgRNA-donor 4.1
GFPpos 29,0
eGFP-sgRNA-donor 4.2
GFPpos 35,0
eGFP-sgRNA-donor 4.3
GFPpos 29,1
eGFP-sgRNA-donor 5.1
GFPpos 0,016
eGFP-sgRNA-donor 5.2
GFPpos 4,30
eGFP-sgRNA-donor 5.3
GFPpos 0,024
eGFP-sgRNA-donor 6.1
GFPpos 1,07
eGFP-sgRNA-donor 6.2
GFPpos 1,83
eGFP-sgRNA-donor 6.3
GFPpos 0,30
GFP
FSC-A

Figure S3. Flow cytometric analysis results for *L. braziliensis* eGFP-Cas9-expressing promastigotes transfected with *eGFP*-targeting sgRNAs.
(A) Gating scheme used to identify the proportion of *L. braziliensis* promastigotes that were GFP-positive.
(B) Representative histogram of GFP expression in the Cas9-eGFP-expressing parental cell line (positive control) compared to an *eGFP*-null mutant (Cas9-derived KO) and Cas9-expressing (GFP-negative) parasites.
(C) Plots showing the percentage of GFP-positive parasites following Cas9-mediated *eGFP* disruption. Six different sets of sgRNAs co-transfected with donor DNAs were tested in triplicate. Pairs of sgRNAs tested are described in the legend of Fig. 1B.

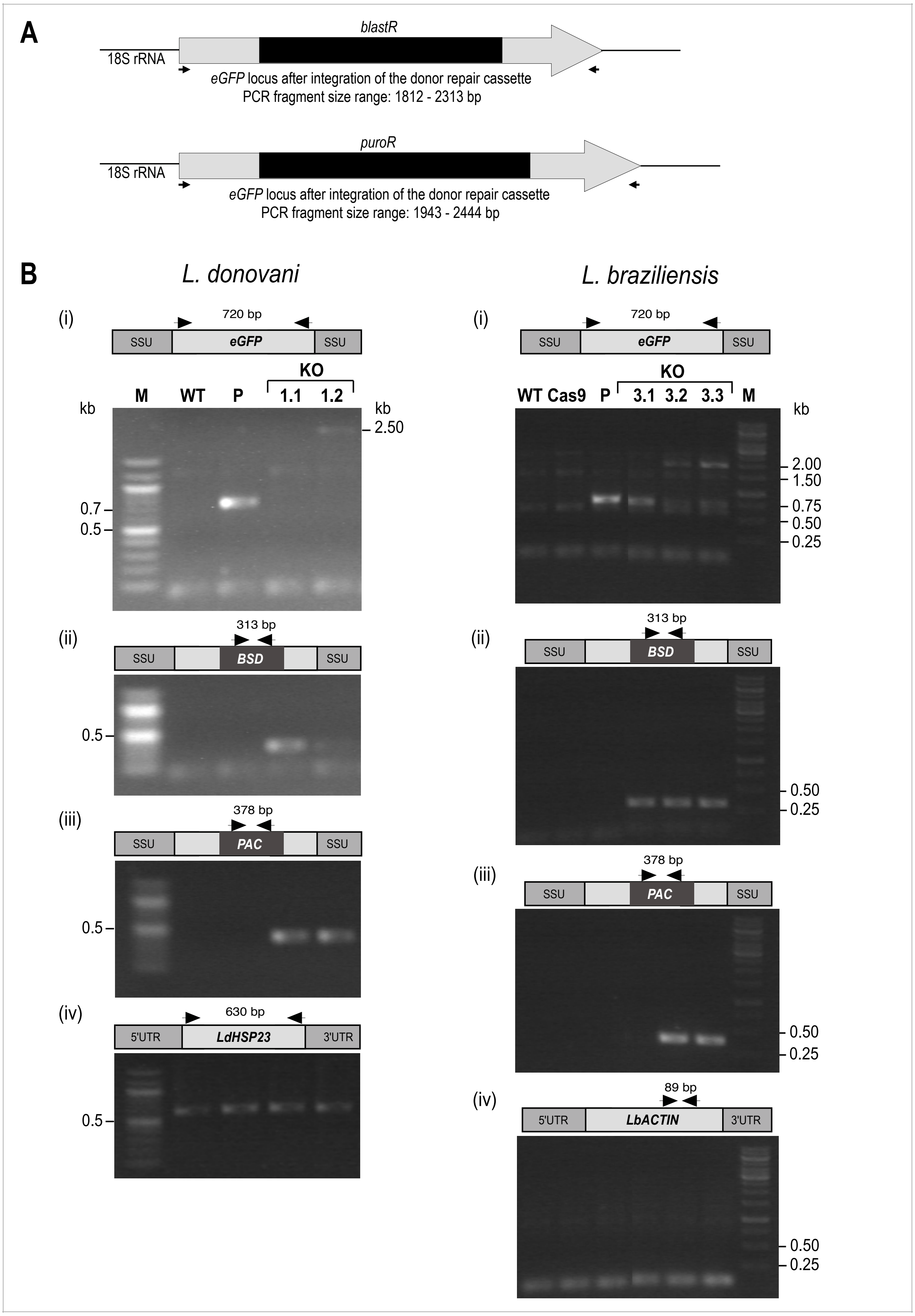

A
blastR
18S rRNA
eGFP locus after integration of the donor repair cassette
PCR fragment size range: 1812 - 2313 bp
puroR
18S rRNA
eGFP locus after integration of the donor repair cassette
PCR fragment size range: 1943 - 2444 bp
B
L. donovani
L. braziliensis
(i)
720 bp
SSU
eGFP
SSU
KO
M WT P 1.1 1.2
kb
2.50
0.7
0.5
WT Cas9 P 3.1 3.2 3.3 M
2.00
1.50
0.75
0.50
0.25
(ii)
313 bp
BSD
(iii)
378 bp
PAC
(iv)
630 bp
5'UTR
LdHSP23
3'UTR
89 bp
LbACTIN

Figure S4. PCR verification of *eGFP* gene replacement. PCR products visualised by agarose gel electrophoresis. Diagrams above the gel pictures show the primers (arrows) used for PCR and size of the expected product. (i) Test for the presence of the *eGFP* locus (integrated into the *Leishmania* SSU rRNA coding region); (ii) test for integration of the blasticidin-resistance gene (*BSD*, Blasticidin-S deaminase); (iii) test for integration of the puromycin-resistance gene (*PAC*, Puromycin N-acetyltransferase); (iv) a parallel PCR reaction amplifying *L. donovani HSP23* ORF or *L. braziliensis actin* ORF was included to verify the presence of DNA.

Left panel: Diagnostic PCR with indicated primers on genomic DNA confirmed the integration of the Blasticidin and Puromycin replacement constructs after sgRNA transfection and absence of the *eGFP* locus in *L. donovani* selected lines. WT, wild-type *L. donovani* 1S strain; P, parental cell line *L. donovani* Cas9/T7/eGFP; KO 1.1 & KO 1.2, *eGFP*-null mutants derived by transfection of *eGFP*-targeting sgRNA-donor DNA set 1. The other *eGFP*-null mutants showed identical results.

Right panel: Diagnostic PCR with indicated primers on genomic DNA showed the presence of the unmodified *eGFP* gene after transfection of *eGFP*-targeting sgRNAs for most *L. braziliensis* selected lines. Since the copy number of the *eGFP* gene integrated in the genome of the *L. braziliensis* Cas9/T7/eGFP parental cell line was not determined, we do not know how many *eGFP* gene copies were efficiently targeted after sgRNA transfection.

While the integration of the Blasticidin replacement construct after transfection was confirmed in all *L. braziliensis* selected lines, there were transfectant cell populations that did not have the Puromycin replacement construct (here shown for KO 3.1), due to the initial moderate selective pressure employed in this species to obtain the intended null mutants. WT, wild-type *L. braziliensis* PER005cl2 strain; Cas9, *L.braz* WT [Cas9]; P, parental cell line *L. braziliensis* Cas9/T7/eGFP; KO 3.1, KO 3.2 & KO 3.3, *eGFP* single-allele mutants derived by transfection of *eGFP*-targeted sgRNA-donor DNA set 3.

M, molecular size marker.

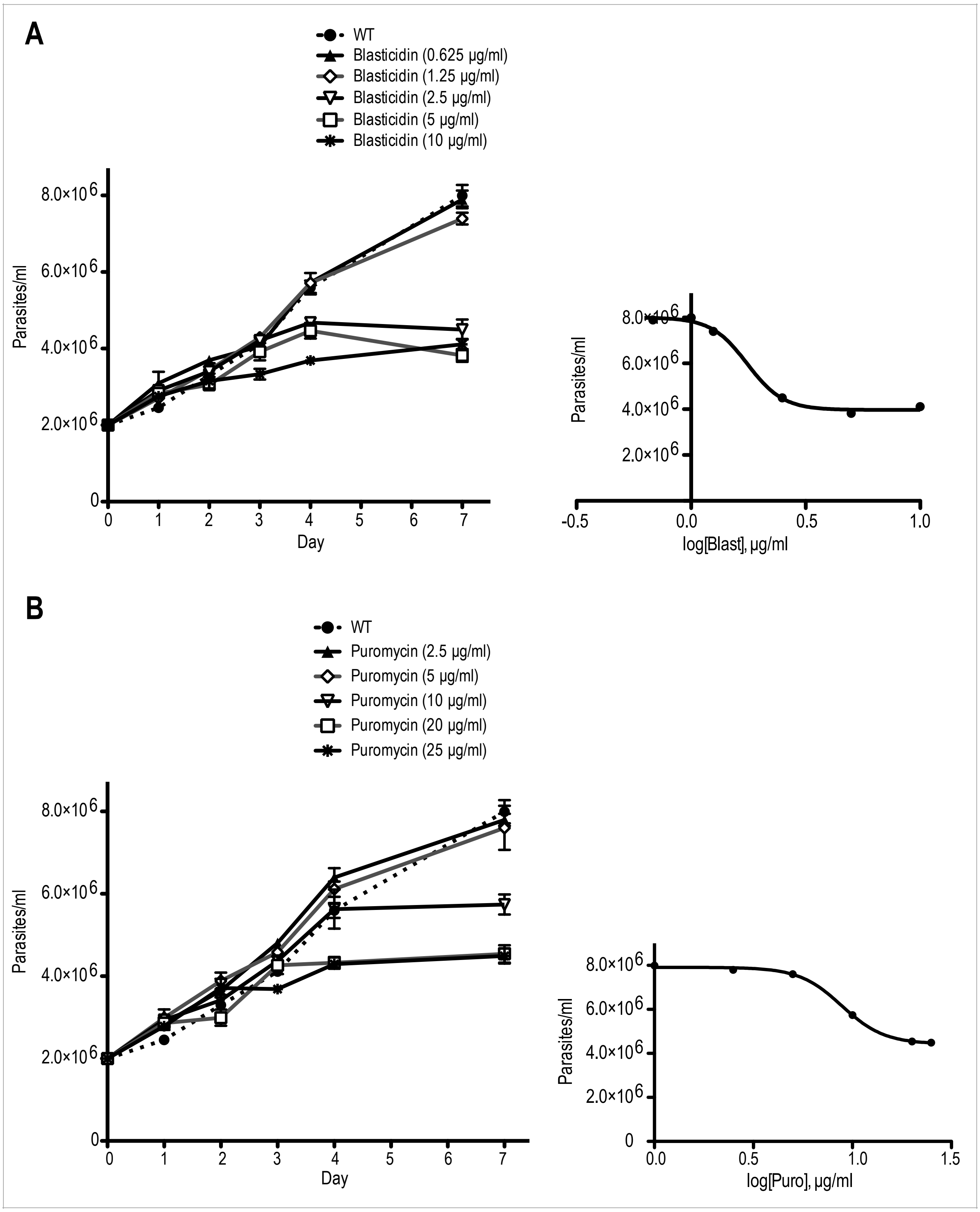
A
WT
Blasticidin (0.625 µg/ml)
Blasticidin (1.25 µg/ml)
Blasticidin (2.5 µg/ml)
Blasticidin (5 µg/ml)
Blasticidin (10 µg/ml)
Parasites/ml
8.0×10^6
6.0×10^6
4.0×10^6
2.0×10^6
0
0
1
2
3
4
5
6
7
Day
Parasites/ml
8.0×10^6
6.0×10^6
4.0×10^6
2.0×10^6
-0.5
0.0
0.5
1.0
log[Blast], µg/ml
B
WT
Puromycin (2.5 µg/ml)
Puromycin (5 µg/ml)
Puromycin (10 µg/ml)
Puromycin (20 µg/ml)
Puromycin (25 µg/ml)
Parasites/ml
8.0×10^6
6.0×10^6
4.0×10^6
2.0×10^6
0
0
1
2
3
4
5
6
7
Day
Parasites/ml
8.0×10^6
6.0×10^6
4.0×10^6
2.0×10^6
0
0.0
0.5
1.0
1.5
log[Puro], µg/ml

Figure S5. Titration of selection antibiotics on *L. braziliensis* promastigotes.
L. braziliensis PER005cl2 WT parasites were seeded at a density of 2×10^6 parasites/ml into 2 ml of complete M199 medium containing a range of increasing concentrations of blasticidin (A) or puromycin (B), and cultured at 25°C for 7 days (in 12-ml cell culture tubes). Note that the highest concentration of antibiotic tested in both cases corresponds to the effective concentrations established for *L. donovani* (1S strain) in our group. The cell density was measured on days 1, 2, 3, 4 and 7 in two biological replicates (each assessed in duplicate) from one experiment. The mean and standard error of the mean (SEM) are shown in the curves (left panel). Cell density data from day 7 were analysed in GraphPad Prism v5.0a to plot dose-response curves and calculate the 50% inhibitory concentration (IC_{50}) using a sigmoidal dose-response model with variable slope (right panel). We used the IC_{50} and the Hill slope parameters of the dose-response curves to calculate the dose of antibiotic necessary to achieve the desired effect.

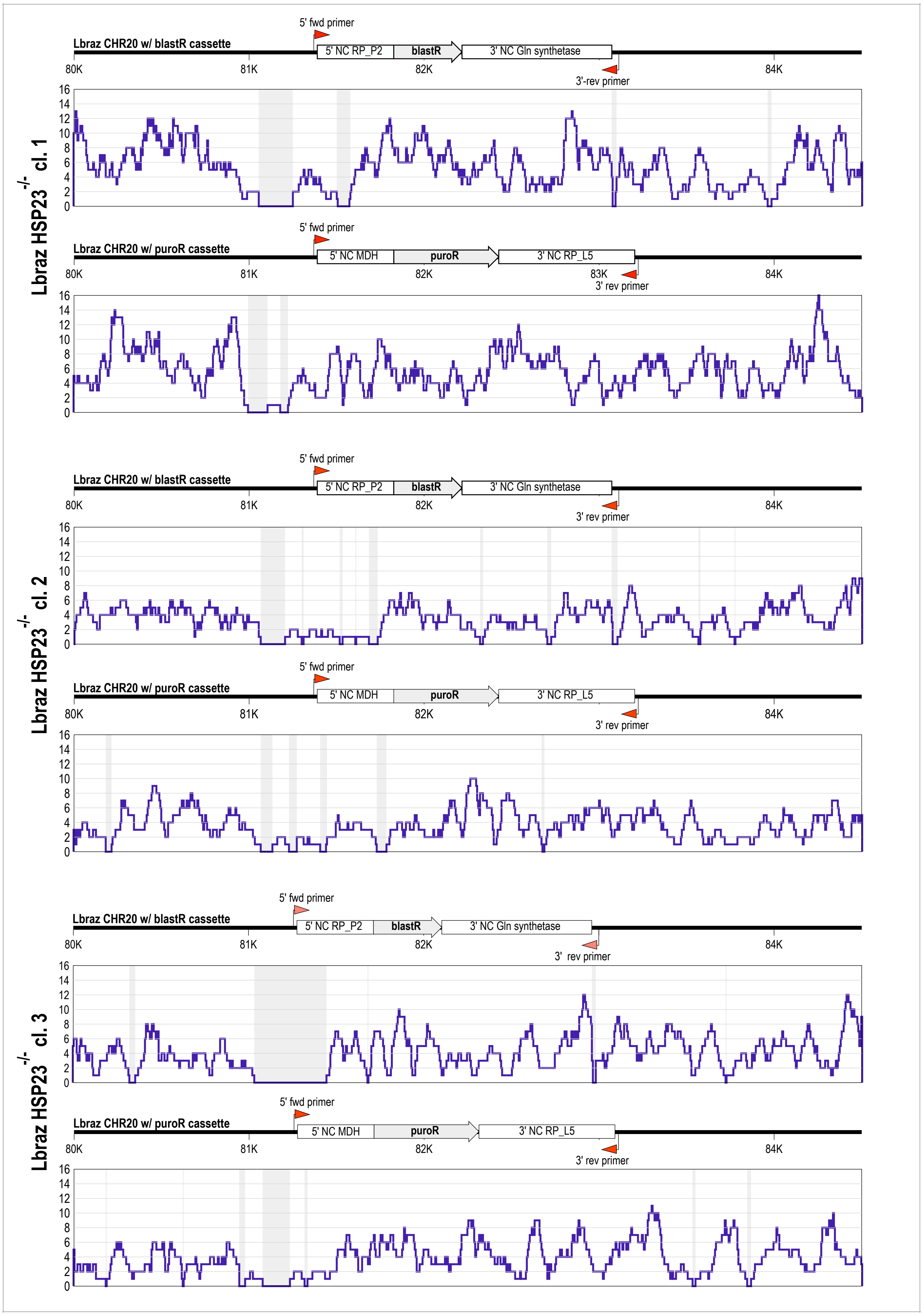
Lbraz HSP23 -/- cl. 1
5' fwd primer
Lbraz CHR20 w/ blastR cassette
5' NC RP_P2
blastR
3' NC Gln synthetase
80K
81K
82K
84K
3'-rev primer
16
14
12
10
8
6
4
2
0
Lbraz CHR20 w/ puroR cassette
5' NC MDH
puroR
3' NC RP_L5
83K
3' rev primer
Lbraz HSP23 -/- cl. 2
Lbraz HSP23 -/- cl. 3

Figure S6. Verification of the *L. braziliensis HSP23* gene replacement by the respective resistance cassettes. Sequence reads from each analysed strain were aligned to the *in silico*-designed reference DNA sequences consisting of the *L. braziliensis* genome (DNA) sequence of chromosome 20 (M2904 reference genome) with the expected insertion of antibiotic resistance cassettes (blastR = blasticidin; PuroR = Puromycin). The Y-axis represents the number of reads and the X-axis shows the nucleotide position (bp) on chromosome 20. Grey shaded areas denote complete lack of aligned reads.

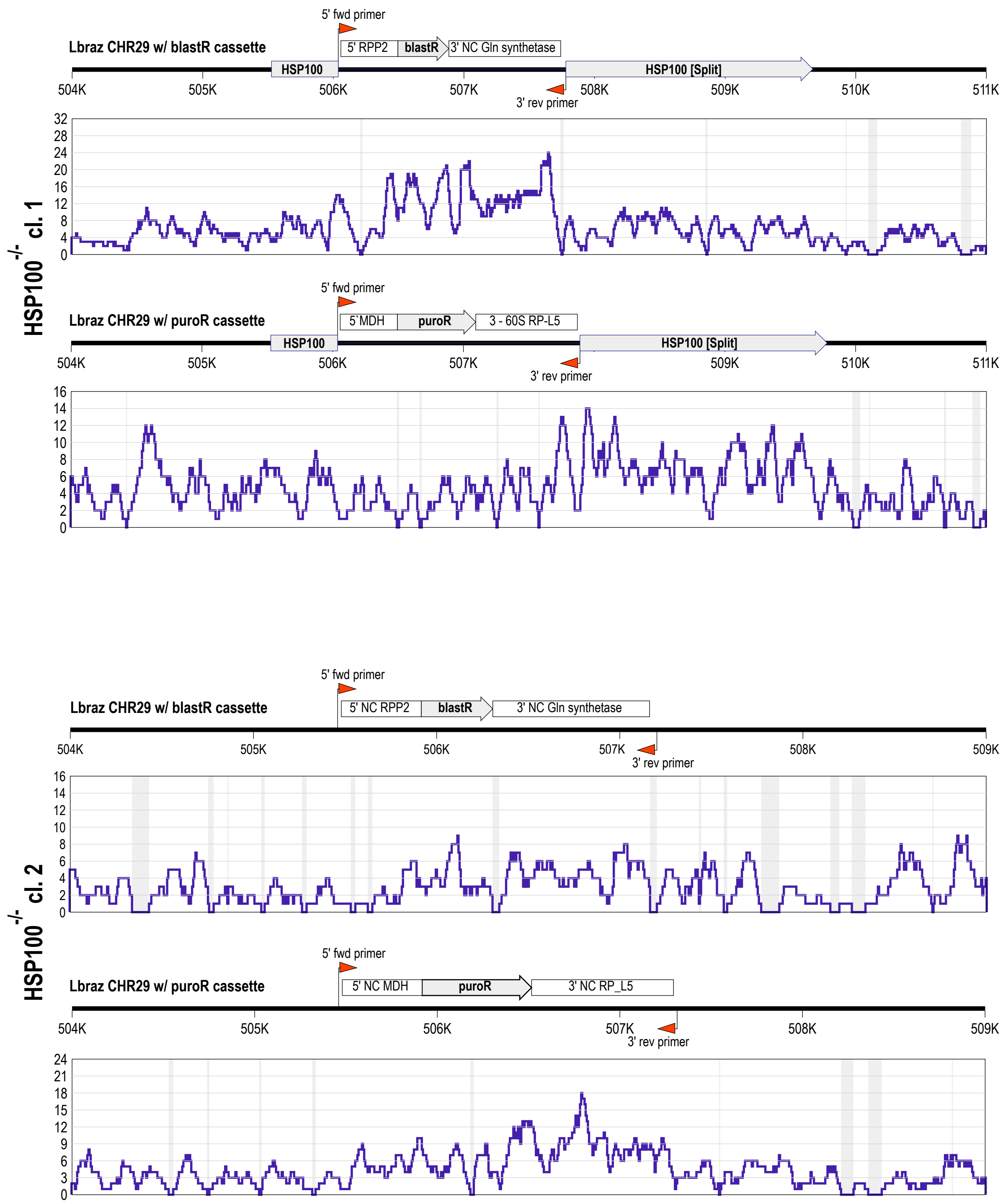

Figure S7. Verification of the *L. braziliensis HSP100* gene replacement by the respective resistance cassettes. Sequence reads from each analysed strain were aligned to the *in silico*-designed reference DNA sequences consisting of the *L. braziliensis* genome (DNA) sequence of chromosome 29 (M2904 reference genome) with the expected insertion of antibiotic resistance cassettes (blastR = blasticidin; PuroR = Puromycin). The Y-axis represents the number of reads and the X-axis shows the nucleotide position (bp) on chromosome 29. Grey shaded areas denote complete lack of aligned reads.

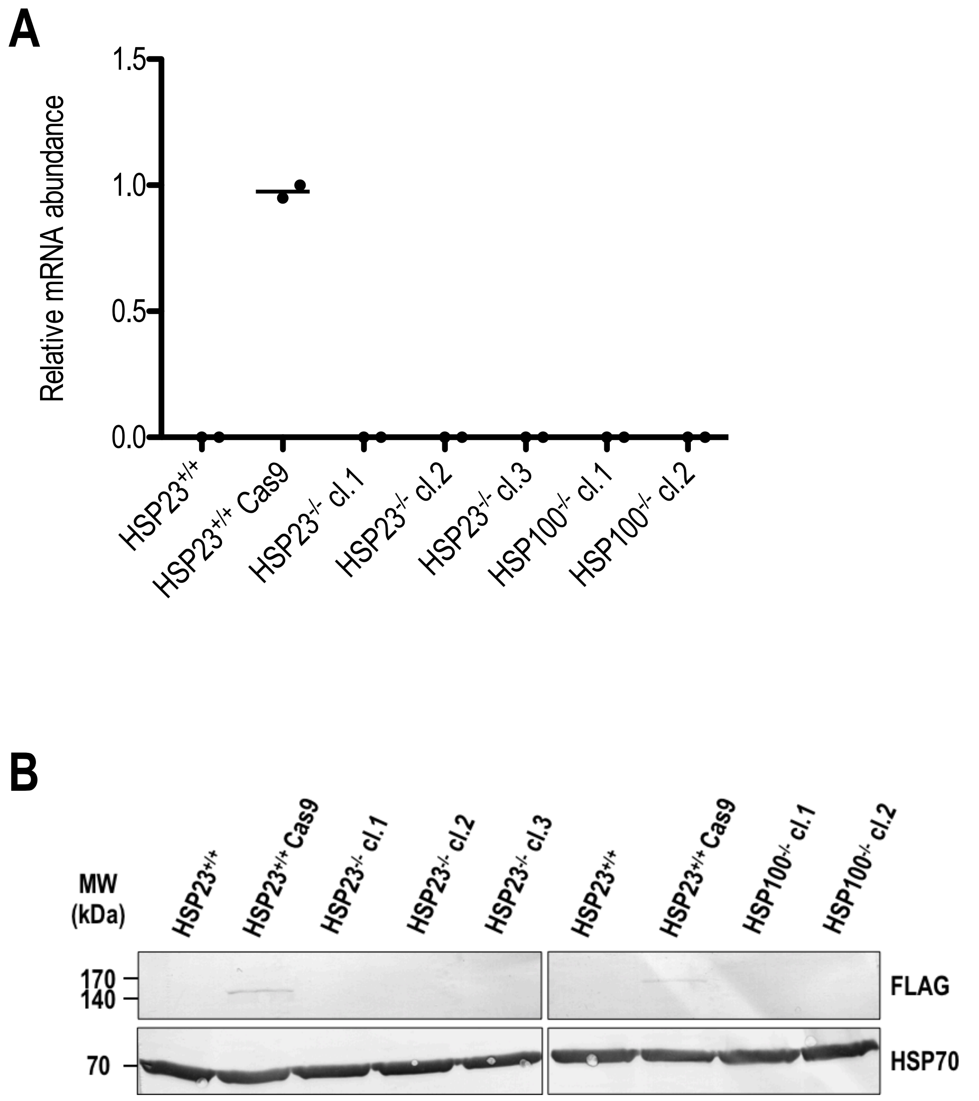

Figure S8. Cas9 expression in *L. braziliensis HSP23*- and *HSP100*-null mutants.
L.braz HSP23$^{+/+}$ WT, *L.braz*:Cas9, and mutant cloned lines *L.braz HSP23*$^{-/-}$ and *L.braz HSP100*$^{-/-}$ were tested for FLAG-tagged Cas9 expression by qRT-PCR using Cas9-specific primers (A) and Western blot using anti-FLAG antibody (1/200). HSP70 antibody (1/500) was used as loading control (B).

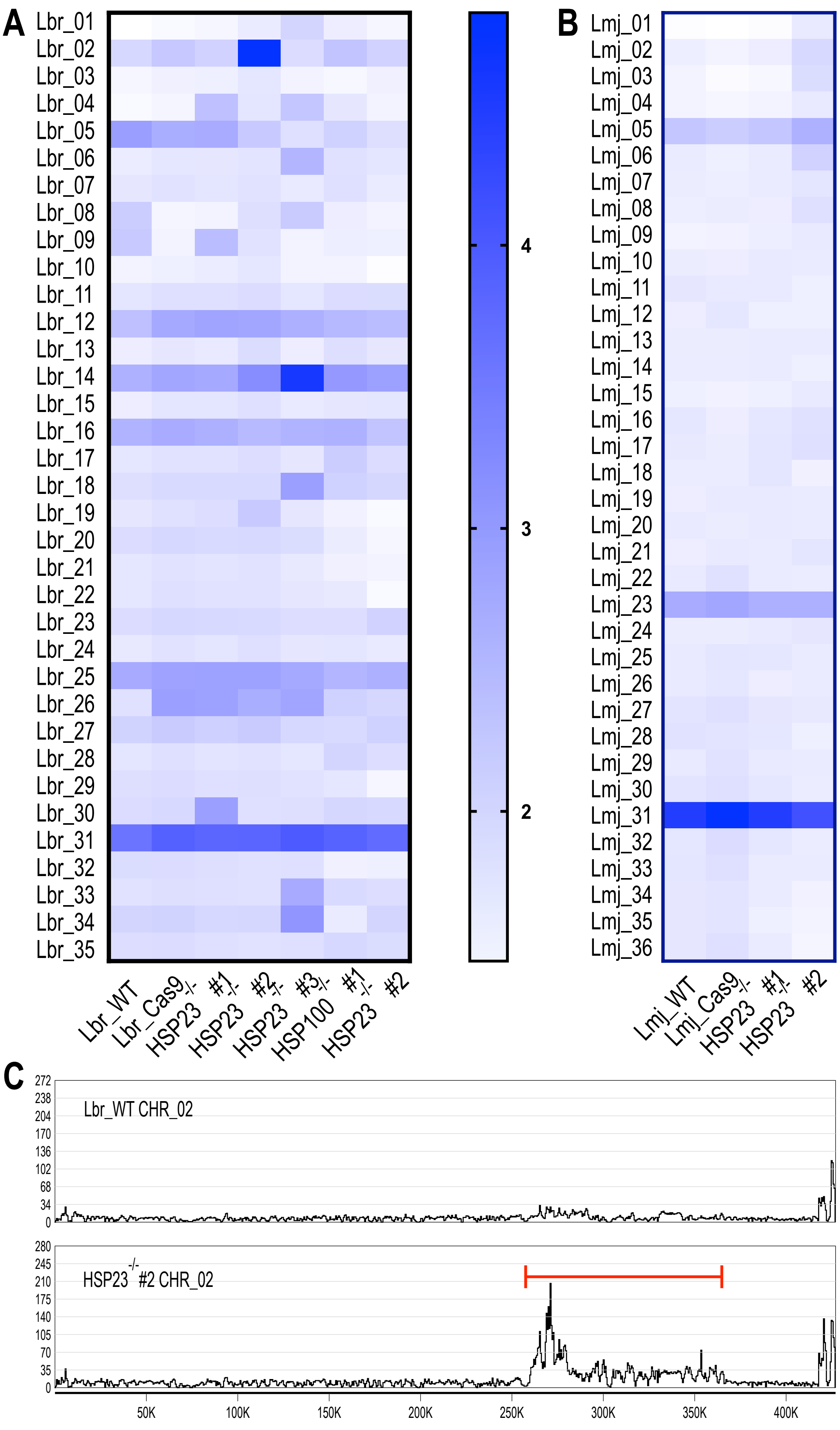

A
Lbr_01
Lbr_02
Lbr_03
Lbr_04
Lbr_05
Lbr_06
Lbr_07
Lbr_08
Lbr_09
Lbr_10
Lbr_11
Lbr_12
Lbr_13
Lbr_14
Lbr_15
Lbr_16
Lbr_17
Lbr_18
Lbr_19
Lbr_20
Lbr_21
Lbr_22
Lbr_23
Lbr_24
Lbr_25
Lbr_26
Lbr_27
Lbr_28
Lbr_29
Lbr_30
Lbr_31
Lbr_32
Lbr_33
Lbr_34
Lbr_35
Lbr_WT
Lbr_Cas9
HSP23 -/- #1
HSP23 -/- #2
HSP23 -/- #3
HSP100 -/- #1
HSP23 -/- #2
4
3
2
B
Lmj_01
Lmj_02
Lmj_03
Lmj_04
Lmj_05
Lmj_06
Lmj_07
Lmj_08
Lmj_09
Lmj_10
Lmj_11
Lmj_12
Lmj_13
Lmj_14
Lmj_15
Lmj_16
Lmj_17
Lmj_18
Lmj_19
Lmj_20
Lmj_21
Lmj_22
Lmj_23
Lmj_24
Lmj_25
Lmj_26
Lmj_27
Lmj_28
Lmj_29
Lmj_30
Lmj_31
Lmj_32
Lmj_33
Lmj_34
Lmj_35
Lmj_36
Lmj_WT
Lmj_Cas9
HSP23 -/- #1
HSP23 -/- #2
C
Lbr_WT CHR_02
272
238
204
170
136
102
68
34
0
HSP23 -/- #2 CHR_02
280
245
210
175
140
105
70
35
0
50K
100K
150K
200K
250K
300K
350K
400K

Figure S9. Karyotype analysis of *L. braziliensis* and *L. major* strains. (A, B) Whole genome sequencing reads were aligned to the *L. braziliensis* or *L. major* reference genome, respectively. Aligned read numbers per chromosome were normalised against total aligned reads per sample and the median aligned read number per 100,000 bp was determined. All aligned read numbers were normalised against the median read density which was set as n=2 and interpreted as chromosome ploidy. (C) Read alignment plot for chromosome 2, comparing reads from *L. braziliensis* WT with *L. braziliensis HSP23*$^{-/-}$ clone #2. The red bar delineates a region of increased read alignment, to be interpreted as amplified gene region. The numbers on the X-axis are the positions (in bp) on the chromosome 2, the numbering on the Y-axis indicates read alignment density.

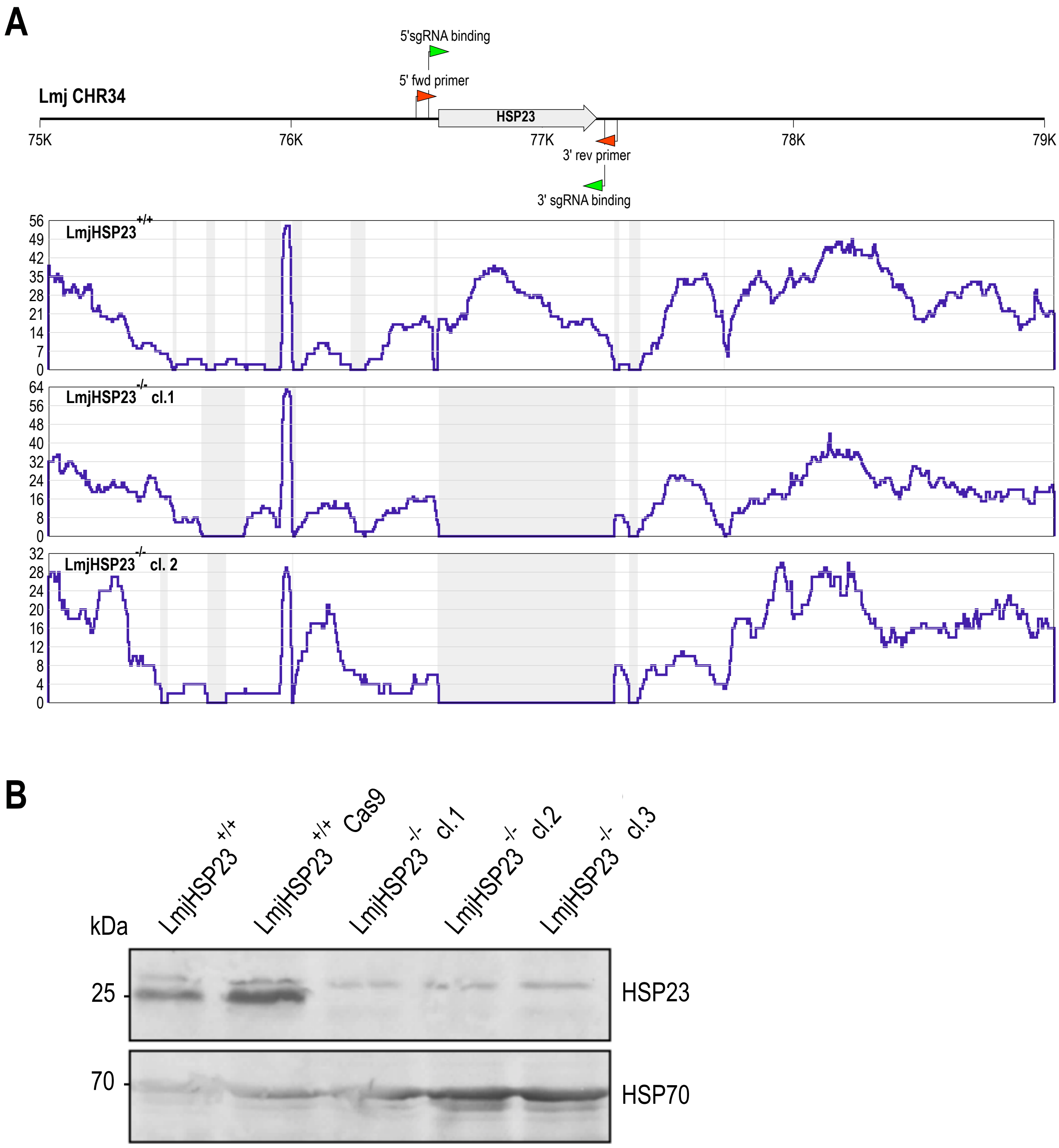

Figure S10. Verification of *L. major HSP23*-null mutants.
Genomic DNA of three *L. major HSP23*$^{-/-}$ clones was isolated and analysed by NGS. Resulting NGS reads were aligned to the *HSP23* gene locus of the *L. major* LV39c5 reference genome (A) using Bowtie 2 software. The read coverages (Y-axis) for the *HSP23* locus are shown in blue. The arrow represents the position and direction of the coding sequence. The X-axis numbering refers to the nucleotide positions (bp) on chromosome 34. A total of $1{\times}10^7$ cells of *L.major* WT, *L.major*:Cas9, and *L.major HSP23*$^{-/-}$ mutants were lysed under denaturing conditions, the cell lysate was separated by SDS-PAGE and subjected to Western blotting using anti-HSP23 (1/500) antibody. HSP70 antibody (1/500) was used as loading control (B).

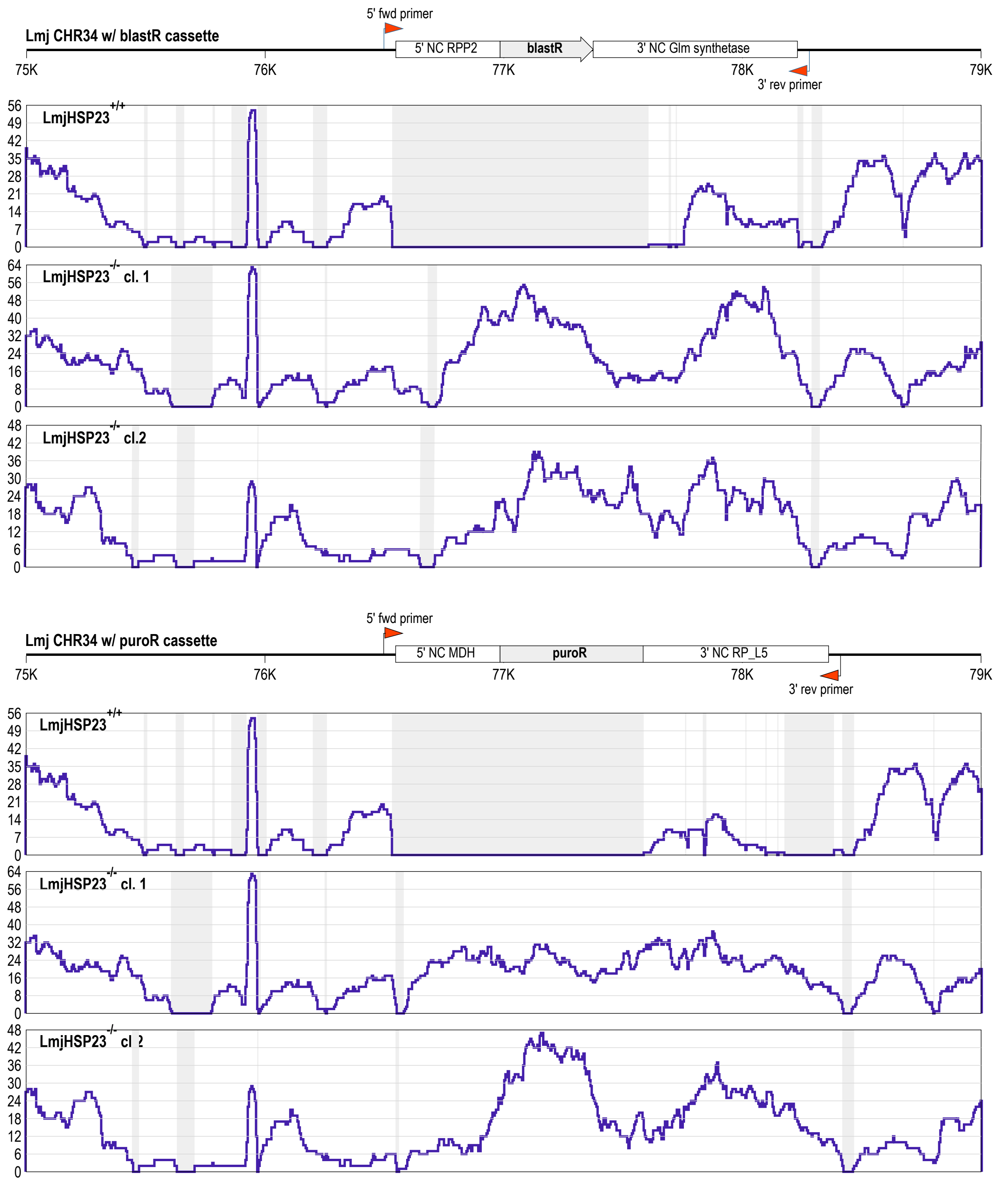

Figure S11. Verification of replacement cassette integration into the *L.major HSP23* locus. Sequence reads from each analysed strain were aligned to the *in silico*-designed reference DNA sequences consisting of the *L. major* genome (DNA) sequence of chromosome 34 (*L. major* LV39c5 reference genome) with the expected insertion of antibiotic resistance cassettes (blastR = blasticidin; PuroR = Puromycin). The Y-axis represents the number of reads and the X-axis shows the nucleotide position (bp) on chromosome 34. Grey shaded areas denote complete lack of aligned reads.

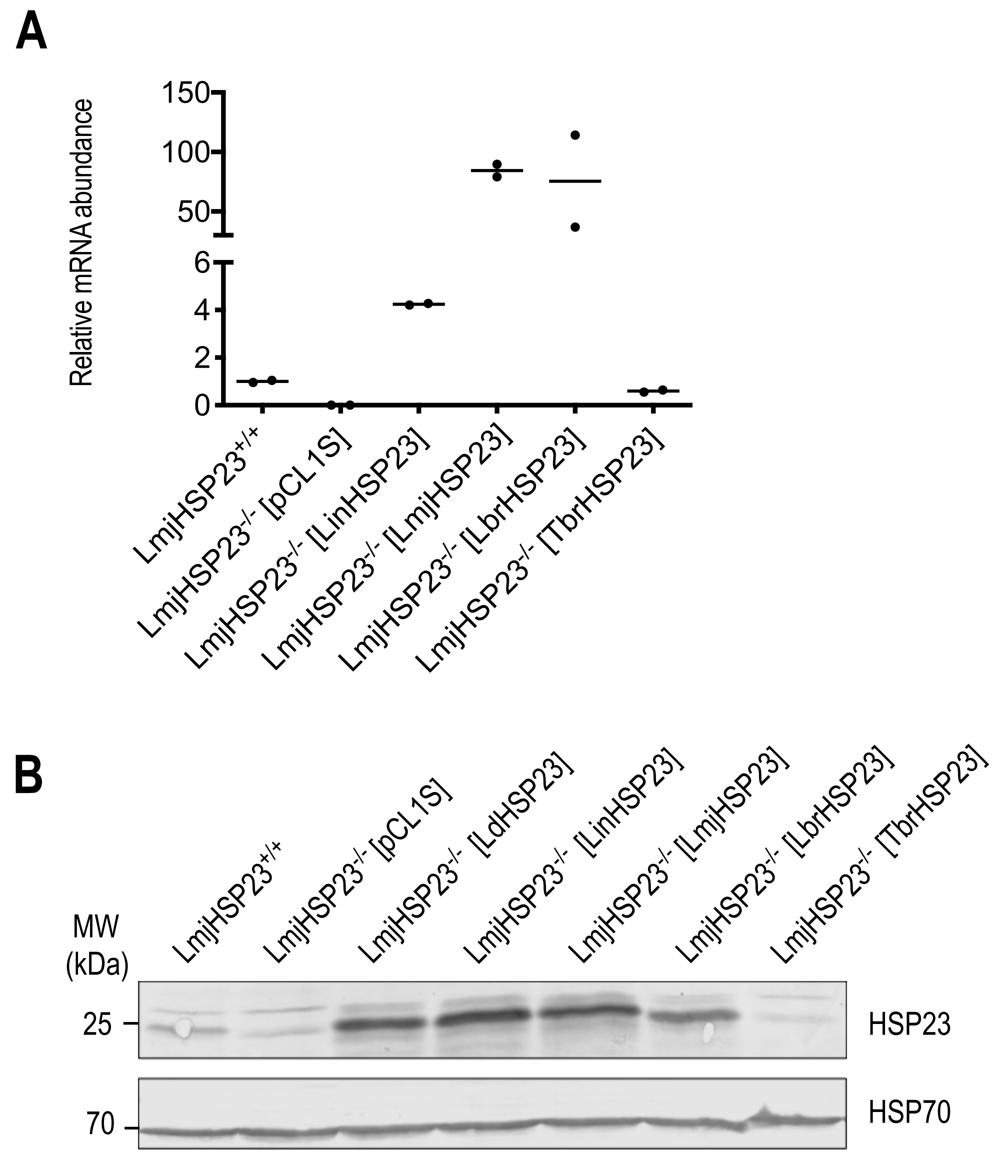

Figure S12. Verification of *L.major HSP23*$^{-/-}$ complementation lines. Over expression of the different HSP23 homologs (*L. donovani*; *L. infantum*; *L. major*; *L. braziliensis*; *T. brucei*) was confirmed on mRNA level by qRT-PCR (A) or by Western blot using antibodies directed against *L. donovani* HSP23. HSP70 serves as loading control (B). Note: the bands seen in the lanes 2 and 7 of the anti-HSP23 Western blot are background.

Table S1. List of primers used in this study for CRISPR-Cas9-mediated gene disruption of *eGFP* and endogenous *HSP23* and *HSP100* genes in *L. braziliensis*, CRISPR-Cas9-mediated gene disruption of *HSP23* and complementation studies in *L. major*, diagnostic PCRs for *eGFP* gene deletion verification, and qRT-PCR measurements.

Name	Sequence 5′ - 3′	Application	Sets of sgRNAs
G00	AAAAGCACCGACTCGGTGCCACTTTTTCAAGTTGATAACGGACTAGCCTTATTTTAACTTGCTATTTCTAGCTCTAAAAC	sgRNA scaffold; for amplification of sgRNA templates	
P9-eGFP-52-5′sgRNA	gaaattaatacgactcactataggGAGCTGGACGGCGACGTAAAgttttagagctagaaatagc	Disruption of *eGFP* (proof-of-principle test)	Retrieved from the Addgene repository; tested in sets 1, 2, 3
P10-eGFP-52-upstream fwd	CTGTTCACCGGGGTGGTGCCCATCCTGGTCgtataatgcagacctgctgc	Disruption of *eGFP* (proof-of-principle test)	
P11-eGFP-553-5′sgRNA	gaaattaatacgactcactataggCAGAACACCCCCATCGGCGAgttttagagctagaaatagc	Disruption of *eGFP* (proof-of-principle test)	Retrieved from the Addgene repository; tested in set 4
P12-eGFP-553-upstream fwd	GGCAGCGTGCAGCTCGCCGACCACTACCAGgtataatgcagacctgctgc	Disruption of *eGFP* (proof-of-principle test)	
P13-eGFP-378-5′sgRNA	gaaattaatacgactcactataggGAAGGGCATCGACTTCAAGGgttttagagctagaaatagc	Disruption of *eGFP* (proof-of-principle test)	Tested in sets 5 and 6
P14-eGFP-378-upstream fwd	GGGCGACACCCTGGTGAACCGCATCGAGCTgtataatgcagacctgctgc	Disruption of *eGFP* (proof-of-principle test)	
P15-eGFP-639-3′sgRNA	gaaattaatacgactcactataggGTTGGGGTCTTTGCTCAGGGgttttagagctagaaatagc	Disruption of *eGFP* (proof-of-principle test)	Tested in sets 3, 4 and 6
P16-eGFP-639-downstream rev	CTCCAGCAGGACCATGTGATCGCGCTTCTCccaatttgagagacctgtgc	Disruption of *eGFP* (proof-of-principle test)	
P17-eGFP-253-3′sgRNA	gaaattaatacgactcactataggAGAAGTCGTGCTGCTTCATGgttttagagctagaaatagc	Disruption of *eGFP* (proof-of-principle test)	Tested in set 1
P18-eGFP-253-downstream rev	GGACGTAGCCTTCGGGCATGGCGGACTTGAccaatttgagagacctgtgc	Disruption of *eGFP* (proof-of-principle test)	
P19-eGFP-612-3′sgRNA	gaaattaatacgactcactataggGGTGCTCAGGTAGTGGTTGTgttttagagctagaaatagc	Disruption of *eGFP* (proof-of-principle test)	Tested in sets 2 and 5
P20-eGFP-612-downstream rev	CTCGTTGGGGTCTTTGCTCAGGGCGGACTGccaatttgagagacctgtgc	Disruption of *eGFP* (proof-of-principle test)	
P33-LbrHSP23-70-5′sgRNA	gaaattaatacgactcactataggATTCCGCTGCAGGACACCACgttttagagctagaaatagc	Partial disruption of *LbrHSP23* in *L.braziliensis*	Tested in set 1
P35-LbrHSP23-377-5′sgRNA	gaaattaatacgactcactataggAAGGTGCTACAGACGACTTGgttttagagctagaaatagc	Partial disruption of *LbrHSP23* in *L.braziliensis*	Tested in set 3
P37-LbrHSP23-183-5′sgRNA	gaaattaatacgactcactataggTTACGGCGCGATATCCTCTGgttttagagctagaaatagc	Partial disruption of *LbrHSP23* in *L.braziliensis*	Tested in set 2

Table S1. List of primers used in this study for CRISPR-Cas9-mediated gene disruption of *eGFP* and endogenous *HSP23* and *HSP100* genes in *L. braziliensis*, CRISPR-Cas9-mediated gene disruption of *HSP23* and complementation studies in *L. major*, diagnostic PCRs for *eGFP* gene deletion verification, and qRT-PCR measurements.

Name	Sequence 5′ - 3′	Application	Sets of sgRNAs
P39-LbrHSP23-171-3′sgRNA	gaaattaatacgactcactataggGCATGCGTACTGCTTGCCTTgttttagagctagaaatagc	Partial disruption of *LbrHSP23* in *L.braziliensis*	Tested in set 1
P41-LbrHSP23-530-3′sgRNA	gaaattaatacgactcactataggAATTCAGGAATCGTGCCGGGgttttagagctagaaatagc	Partial disruption of *LbrHSP23* in *L.braziliensis*	Tested in set 3
P43-LbrHSP23-323-3′sgRNA	gaaattaatacgactcactataggACCTTCTCCTTGGTCAGCCGgttttagagctagaaatagc	Partial disruption of *LbrHSP23* in *L.braziliensis*	Tested in set 2
P34-LbrHSP23-70-upstream fwd	GCACAGCGCCCCGAGTATGTCCTCGTCACGgtataatgcagacctgctgc	Partial disruption of *LbrHSP23* in *L.braziliensis*	
P36-LbrHSP23-377-upstream fwd	ACTGGTCTAAGTGGAAAGACGAGGATGACGgtataatgcagacctgctgc	Partial disruption of *LbrHSP23* in *L.braziliensis*	
P38-LbrHSP23-183-upstream fwd	AGGCAAGCAGTACGCATGCACGATCCACTTgtataatgcagacctgctgc	Partial disruption of *LbrHSP23* in *L.braziliensis*	
P40-LbrHSP23-171-downstream rev	AGAGGATATCGCGCCGTAAAAGTGGATCGTccaatttgagagacctgtgc	Partial disruption of *LbrHSP23* in *L.braziliensis*	
P42-LbrHSP23-530-downstream rev	TGAGTGGCCTCTTGCCTTTGCGCCGAGCCAccaatttgagagacctgtgc	Partial disruption of *LbrHSP23* in *L.braziliensis*	
P44-LbrHSP23-323-downstream rev	GACCAGTCGATGGTAATGTTAGGGTATTTGccaatttgagagacctgtgc	Partial disruption of *LbrHSP23* in *L.braziliensis*	
P1-LbrHsp23 - upstream fwd	GTCCACTTGGTCCCCACCCCCACCCGCCCAgtataatgcagacctgctgc	Complete disruption of *LbrHSP23* in *L.braziliensis*	
P45-LbrHSP23-1139-5′sgRNAorig	gaaattaatacgactcactataggTGCACTCAACTGAGCAGGTGgttttagagctagaaatagc	Complete disruption of *LbrHSP23* in *L.braziliensis*	Tested in set 4
P3-LbrHsp23 - downstream rev	TCACGAGACGCAGATCGAAAGAGTGTGTGAccaatttgagagacctgtgc	Complete disruption of *LbrHSP23* in *L.braziliensis*	
P4-LbrHsp23 - 3′sgRNA	gaaattaatacgactcactataggCAAAGAGAAAAGACCCGGTAgttttagagctagaaatagc	Complete disruption of *LbrHSP23* in *L.braziliensis*	Tested in set 4
P21-LbrHSP100-105-5′sgRNA	gaaattaatacgactcactataggCCTCGCCTACACCATGTTTGgttttagagctagaaatagc	Partial disruption of *LbrHSP100* in *L.braziliensis*	Tested in set 2
P23-LbrHSP100-45-5′sgRNA	gaaattaatacgactcactataggGATGGCCCGCACCGTCGCACgttttagagctagaaatagc	Partial disruption of *LbrHSP100* in *L.braziliensis*	Tested in set 1
P25-LbrHSP100-513-5′sgRNA	gaaattaatacgactcactataggGGAGGACGGAAAGCTGGACCgttttagagctagaaatagc	Partial disruption of *LbrHSP100* in *L.braziliensis*	Tested in set 3
P27-LbrHSP100-623-3′sgRNA	gaaattaatacgactcactataggCCAGGTTCACCAATCAGTACgttttagagctagaaatagc	Partial disruption of *LbrHSP100* in *L.braziliensis*	Tested in set 2

**Table S1. List of primers used in this study** for CRISPR-Cas9-mediated gene disruption of *eGFP* and endogenous *HSP23* and *HSP100* genes in *L. braziliensis*, CRISPR-Cas9-mediated gene disruption of *HSP23* and complementation studies in *L. major*, diagnostic PCRs for *eGFP* gene deletion verification, and qRT-PCR measurements.			
**Name**	Sequence 5′ – 3′	**Application**	**Sets of sgRNAs**
P29-LbrHSP100-263-3′s gRNA	gaaattaatacgactcactataggATCATGTCCGAATTGGGCCGgttttagagctagaaatagc	Partial disruption of *LbrHSP100* in *L.braziliensis*	Tested in set 1
P31-LbrHSP100-712-3′s gRNA	gaaattaatacgactcactataggAGAATATGCGAATGCCGTCAgttttagagctagaaatagc	Partial disruption of *LbrHSP100* in *L.braziliensis*	Tested in set 3
P22-LbrHSP100-105-upstream fwd	GAAGGCAAACGGTTATCTCCACCCCGCGCAgtataatgcagacctgctgc	Partial disruption of *LbrHSP100* in *L.braziliensis*	
P24-LbrHSP100-45-upstream fwd	GCCAGAATGGACTCAGGCGGCCTCTGAGTTgtataatgcagacctgctgc	Partial disruption of *LbrHSP100* in *L.braziliensis*	
P26-LbrHSP100-513-upstream fwd	CAAGTACGCAATTGATCTGTGCAAGCAGGCgtataatgcagacctgctgc	Partial disruption of *LbrHSP100* in *L.braziliensis*	
P28-LbrHSP100-623-downstream rev	ATGCCCTCCACAATCGCAGTCTTACCAACTccaatttgagagacctgtgc	Partial disruption of *LbrHSP100* in *L.braziliensis*	
P30-LbrHSP100-263-downstream rev	CGCTCCTGCTCCGCCGTGTTCAGCACGCGCccaatttgagagacctgtgc	Partial disruption of *LbrHSP100* in *L.braziliensis*	
P32-LbrHSP100-712-downstream rev	CACCGGCGACCAGCGCACCCATATCCAGCGccaatttgagagacctgtgc	Partial disruption of *LbrHSP100* in *L.braziliensis*	
P5-LbrHsp100 - upstream fwd	TTGGGTGACTTCGATTAGAATACTGACATAgtataatgcagacctgctgc	Complete disruption of *LbrHSP100* in *L.braziliensis*	
P6-LbrHsp100 - 5′sgRNA	gaaattaatacgactcactataggCCCACGAGAAGAAAAGCCGAgttttagagctagaaatagc	Complete disruption of *LbrHSP100* in *L.braziliensis*	Tested in set 4
P7-LbrHsp100 - downstream rev	TGTCTGCCGTCACTCAGCTCGTCTTCGCTTccaatttgagagacctgtgc	Complete disruption of *LbrHSP100* in *L.braziliensis*	
P8-LbrHsp100 - 3′sgRNA	gaaattaatacgactcactataggCGTCTCTCCATATCCATTGGgttttagagctagaaatagc	Complete disruption of *LbrHSP100* in *L.braziliensis*	Tested in set 4
EGFP-5′Kpn1 fwd	GGGGGTACCGGCAAGCTTACCATGGTGAGCAAGGGCGAGGAG	Diagnostic PCR for *eGFP* knockout verification	
EGFP-3′BamH1 rev	GGGGGATCCTTACTTGTACAGCTCGTC	Diagnostic PCR for *eGFP* knockout verification	
Lbr-actin-F2	CGGCTTCCCAGAGATGGTGTATCAG	Technical control PCR; and reference gene for normalisation of qRT-PCR data (*L.braziliensis* actin)	
Lbr-actin-B2	CGCCAGACAGGACAATGTTGCC	Technical control PCR; and reference gene for normalisation of qRT-PCR data (*L.braziliensis* actin)	

Table S1. List of primers used in this study for CRISPR-Cas9-mediated gene disruption of *eGFP* and endogenous *HSP23* and *HSP100* genes in *L. braziliensis*, CRISPR-Cas9-mediated gene disruption of *HSP23* and complementation studies in *L. major*, diagnostic PCRs for *eGFP* gene deletion verification, and qRT-PCR measurements.

Name	Sequence 5' – 3'	Application	Sets of sgRNAs
p23(2)_genspez_fwd	ATGTCCACCAGCGGCCCA	Technical control PCR (*L. donovani HSP23*)	
p23(2)_genspez_rev	TCACGTGTCCTCCTCGAG	Technical control PCR (*L. donovani HSP23*)	
CRISPR-Puro-fwd	GGACATTGGTAAGGTTTGGGTTG	Diagnostic PCR to test for integration of the Puromycin replacement construct in candidate *eGFP* null mutants	
CRISPR-Puro-rev	GGCACTTCAACATCTGCTGTTAC	Diagnostic PCR to test for integration of the Puromycin replacement construct in candidate *eGFP* null mutants	
CRISPR-Blast-fwd	GAATCCACCCTCATTGAAAGAGC	Diagnostic PCR to test for integration of the Blasticidin replacement construct in candidate *eGFP* null mutants	
CRISPR_blast-rev	CATCACTGTCCTTCACTATCGCT	Diagnostic PCR to test for integration of the Blasticidin replacement construct in candidate *eGFP* null mutants	
P59-Cas9-qPCR_fwd	CGAGAAGCTGTACCTGTACTACC	qRT-PCR to quantify relative mRNA abundance of *Cas9* in *L.brHSP23 & L.brHSP100* null mutants	
P60-Cas9-qPCR_rev	CACGATATGGTCCACATCGTAGT	qRT-PCR to quantify relative mRNA abundance of *Cas9* in *L.brHSP23 & L.brHSP100* null mutants	
T7RNApol_F	TGAGCATGAGTCTTACGAGATGG	qRT-PCR to quantify relative mRNA abundance of *T7 RNAP* in *L.braziliensis* Cas9 T7 parental cell line	
T7RNApol_R	AGTAGGGTAGTGATGAGAGGCTT	qRT-PCR to quantify relative mRNA abundance of *T7 RNAP* in *L.braziliensis* Cas9 T7 parental cell line	
LmHSP23-UFP	CCCGTACACGCCCGCGCGTTTTGGTGCAGCgtataatgcagacctgctgc	Complete disruption of *HSP23* in *L.major*	
LmHSP23-5'SgRNAprimer	gaaattaatacgactcactataggCTGTCTCTGACATTGACGCTgttttagagctagaaatagc	Complete disruption of *HSP23* in *L.major*	
LmHSP23-DRP	TGGCGTCGCTGCAGCGGCAGAGCGCCGCCAccaatttgagagacctgtgc	Complete disruption of *HSP23* in *L.major*	

Table S1. List of primers used in this study for CRISPR-Cas9-mediated gene disruption of *eGFP* and endogenous *HSP23* and *HSP100* genes in *L. braziliensis*, CRISPR-Cas9-mediated gene disruption of *HSP23* and complementation studies in *L. major*, diagnostic PCRs for *eGFP* gene deletion verification, and qRT-PCR measurements.

Name	Sequence 5′ - 3′	Application	Sets of sgRNAs
LmHSP23-3′SgRNAprimer	gaaattaatacgactcactataggAAGCCTGCTCAATCGACCTGgttttagagctagaaatagc	Complete disruption of *HSP23* in *L.major*	
LbrM_HSP23_BclI_rev	GGAGTGATCATCACATATCGTCCTCGAGCG	complementation studies	
LbrM_HSP23_KpnI_fwd	GGAGGGTACCATGTCGGCCAGCGGTTCATTG	complementation studies	
Lmaj_HSP23_BclI_rev	GGAGTGATCATCACGTGTCCTCCTCGAGCG	complementation studies	
Lmaj_HSP23_KpnI_fwd	GGAGGGTACCATGTCCAGCAGCGGCCCATTG	complementation studies	
Tb_HSP23_BclI_rev	GGAGTGATCATTACGGTTCGAGAGGAGGCAG	complementation studies	
Tb_HSP23_KpnI_fwd	GGAGGGTACCATGACGAATCAGTCGGACGC	complementation studies	
p23.2_5′KpnI	GGGGGTACCATGTCCACCAGCGGCCCATTG	complementation studies	
p23.2_3′BamHI	GGGGGATCCTCACGTGTCCTCCTCGAGC	complementation studies	
LmHSP23_qPCR_fwd	CGGAAGAAGTTCTCCAAGTCACT	qRT-PCR to quantify relative mRNA abundance of *L.major HSP23*	
LmHSP23_qPCR_rev	GTCTTTCCACTTAGACCAGTCGA	qRT-PCR to quantify relative mRNA abundance of *L.major HSP23*	
Lbr_HSP23_qPCR_fwd	CTGCGGAAAAAGCTAACAAGGTC	qRT-PCR to quantify relative mRNA abundance of *L.braziliensis HSP23*	
Lbr_HSP23_qPCR_rev	CGTCATCCTCGTCTTTCCACTTA	qRT-PCR to quantify relative mRNA abundance of *L.braziliensis HSP23*	
Tbruc_HSP23_qPCR_fwd	GAATCAGTCGGACGCGATATTTC	qRT-PCR to quantify relative mRNA abundance of *T.brucei HSP23*	
Tbruc_HSP23_qPCR_rev	CTTTATCTCCACCACAACGTTCG	qRT-PCR to quantify relative mRNA abundance of *T.brucei HSP23*	

Table S1. List of primers used in this study for CRISPR-Cas9-mediated gene disruption of *eGFP* and endogenous *HSP23* and *HSP100* genes in *L. braziliensis*, CRISPR-Cas9-mediated gene disruption of *HSP23* and complementation studies in *L. major*, diagnostic PCRs for *eGFP* gene deletion verification, and qRT-PCR measurements.

Name	Sequence 5′ - 3′	Application	Sets of sgRNAs
p23.2-F42	CCATTCCTGGATTTGGCTCGG	qRT-PCR to quantify relative mRNA abundance of *L.donovani HSP23*	
p23.2-B36	GCATATCGCCGTCGTCATCTCC	qRT-PCR to quantify relative mRNA abundance of *L.donovani HSP23*	

Table S2. Sequence identity analysis of trypanosomatid HSP23 proteins. Amino acid sequences of the different HSP23 orthologs were aligned by CLUSTALW using the MacVector Software. Sequence identity list shown in %.

	L. donovani	*L. infantum*	*L. major*	*L. braziliensis*	*T. brucei*
L. donovani	100.0				
L. infantum	99.5	100.0			
L. major	95.2	94.7	100.0		
L. braziliensis	85.2	85.2	84.2	100.0	
T. brucei	36.3	36.3	37.3	36.3	100.0
